# Free serum haemoglobin is associated with brain atrophy in secondary progressive multiple sclerosis

**DOI:** 10.12688/wellcomeopenres.9967.2

**Published:** 2016-12-23

**Authors:** Alex Lewin, Shea Hamilton, Aviva Witkover, Paul Langford, Richard Nicholas, Jeremy Chataway, Charles R.M. Bangham

**Affiliations:** 1 Department of Epidemiology and Biostatistics, Imperial College London, London, UK; 2 Division of Infectious Diseases, Department of Medicine, Imperial College London, London, UK; 3 Division of Brain Sciences, Department of Medicine, Imperial College London, London, UK; 4 National Hospital for Neurology and Neurosurgery, University College London Hospitals NHS Foundation Trust and Queen Square Multiple Sclerosis Centre, Department of Neuroinflammation, University College London, London, UK; 5 Present address: Department of Mathematics, Brunel University, London, UK

**Keywords:** Secondary progressive multiple sclerosis, neurodegeneration, brain atrophy, iron, haemoglobin, proteomics, pathogenesis

## Abstract

**Background**: A major cause of disability in secondary progressive multiple sclerosis (SPMS) is progressive brain atrophy, whose pathogenesis is not fully understood. The objective of this study was to identify protein biomarkers of brain atrophy in SPMS.

**Methods**: We used surface-enhanced laser desorption-ionization time-of-flight mass spectrometry to carry out an unbiased search for serum proteins whose concentration correlated with the rate of brain atrophy, measured by serial MRI scans over a 2-year period in a well-characterized cohort of 140 patients with SPMS.  Protein species were identified by liquid chromatography-electrospray ionization tandem mass spectrometry.

**Results**: There was a significant (p<0.004) correlation between the rate of brain atrophy and a rise in the concentration of proteins at 15.1 kDa and 15.9 kDa in the serum.  Tandem mass spectrometry identified these proteins as alpha-haemoglobin and beta-haemoglobin, respectively.  The abnormal concentration of free serum haemoglobin was confirmed by ELISA (p<0.001).  The serum lactate dehydrogenase activity was also highly significantly raised (p<10 ^-12^) in patients with secondary progressive multiple sclerosis.

**Conclusions**: The results are consistent with the following hypothesis. In progressive multiple sclerosis, low-grade chronic intravascular haemolysis releases haemoglobin into the serum; the haemoglobin is subsequently translocated into the central nervous system (CNS) across the damaged blood-brain barrier.  In the CNS, the haemoglobin and its breakdown products, including haem and iron, contribute to the neurodegeneration and consequent brain atrophy seen in progressive disease. We postulate that haemoglobin is a source of the iron whose deposition along blood vessels in multiple sclerosis plaques is associated with neurodegeneration.  If so, then chelators of haemoglobin, rather than chelators of free serum iron, may be effective in preventing this neurodegeneration.

## Introduction

In multiple sclerosis (MS), progressive disease develops in over half of those who present with an initial relapsing phase – secondary progressive MS (SPMS) – but can also present as primary progressive MS (PPMS). Progressive MS, for which there is no clear disease-modifying treatment ^1– 3^, accounts for much of the disability and the cost of MS to both the person and the community ^4^.

Unlike relapsing-remitting MS (RRMS), where an inflammatory response involving the adaptive immune system leads to episodic neurological deficits, in progressive MS neuroaxonal loss leads to an increasing neurological deficit and brain atrophy ^5, 6^. However, in all forms of the disease, both the initiating events and the mechanisms of pathogenesis remain uncertain ^5^. Pseudoatrophy may account for some loss of brain volume ^7^, but brain atrophy has also been associated with changes in neurofilament levels ^8^ and sodium metabolism ^9^.

The objective of the present study was to use an unbiased, high-throughput technique to identify protein biomarkers of brain atrophy in a longitudinal cohort of patients with SPMS. We used surface-enhanced laser desorption-ionization time-of-flight (SELDI-TOF) mass spectrometry to analyse serial serum samples from the population that participated in the MS-STAT study (described below) ^1^, to identify proteins whose abundance was associated with MRI-measured brain atrophy rate. Serum proteomics in MS has previously been investigated in small cross-sectional studies to compare relapsing MS and progressive disease ^10– 12^. However, these previous studies were neither designed nor powered to identify correlates of neurodegeneration in SPMS.

We found that the rate of brain atrophy in this cohort was associated with an increase in the concentration of free haemoglobin in the serum. This association was independent of the beneficial effect of simvastatin treatment, which remained significant in the present analysis. An ELISA assay confirmed the presence of abnormal concentrations of free haemoglobin in the serum of patients with SPMS. In addition, the serum lactate dehydrogenase (LDH) activity was significantly greater in patients with SPMS than in three different groups of control subjects. These results suggest that chronic intravascular haemolysis releases haemoglobin into the serum in SPMS; we postulate that this haemoglobin is a source of the abnormal iron deposition along blood vessels in the central nervous system that is associated with neurodegeneration in progressive MS.

## Methods

### Ethical approval

The study was done in accordance with Good Clinical Practice and the Declaration of Helsinki. The protocol was approved by the UK National Research Ethics Service (Berkshire Research Ethics Committee; reference 07/Q1602/73), and every patient gave written informed consent before entering the study.

### Subjects

The MS-STAT clinical trial was registered with ClinicalTrials.gov, number NCT00647348, and has been described in detail elsewhere ^1^. In this phase 2 placebo-controlled double-blind trial, 140 patients with SPMS were randomized 1:1 to simvastatin 80 mg/day (40 mg for the first month) or matched placebo. The patients were in trial for 2 years. The primary outcome was change in whole brain volume as measured by the Brain Boundary Shift Integral (BBSI), with MRI data acquired at baseline, 12 months and 25 months; the last MRI scan (25 months) was carried out 1 month after last medication to minimize any potential artefactual changes in volume ^1^. Simvastatin treatment resulted in a highly statistically significant 43% reduction in the annualized rate of brain atrophy ^1^, and significant changes were also seen in certain clinician- and patient-reported outcome measures. As control groups in the haemoglobin assays, we studied healthy adult volunteers (n=20); patients with human T-lymphotropic virus (HTLV-1)-associated myelopathy/tropical spastic paraparesis (HAM/TSP), which closely resembles chronic spinal forms of multiple sclerosis (n=20); and asymptomatic HTLV-1 carriers (n=20).

### Protein profiling of serum by SELDI-TOF mass spectrometry

Serum samples were collected and cryopreserved from each patient at baseline, 6 months, 12 months and 24 months. Samples were not available from all patients at each time-point; a total of 475 samples were available for SELDI-TOF mass spectrometry. Samples were randomized, and staff were blinded to the treatment arms. CM10 ProteinChip arrays (Bio-Rad Laboratories) were primed with binding buffer (50 mM ammonium acetate, 0.01% Triton X-100, pH 4·0) and incubated at room temperature (RT) for 5 min. A 1:10 dilution of serum in binding buffer was then applied to the array and incubated at RT for 1 hr. The arrays were washed twice with binding buffer and deionized water. Saturated sinapinic acid (0.7 µL) was applied twice to each spot on the arrays. Time-of-flight spectra were generated using a PCS-4000 mass spectrometer (Bio-Rad). Low-range spectra (mass/charge (m/z) ratio 0 – 20,000) were obtained at a laser energy of 3000 nJ, with a focus mass of 6000 and the matrix attenuated to 1000. High-range spectra (m/z 10,000 – 75,000) were obtained at a laser energy of 3900 nJ, with a focus mass of 30,000 and the matrix attenuated to 10,000. Mass accuracy was calibrated externally using All-in-One Peptide or Protein molecular mass standards (Bio-Rad).

### Proteomics data processing

Spectra were analysed using ProteinChip Data Manager (Bio-Rad version 4.1.0) and normalized using total ion current. Peaks were auto-detected using a peak threshold of 12.5% and a mass window of 0.3%, and the resulting data were converted for subsequent analysis using R software. The abundance (intensity) of a given protein peak was quantified as the area under the peak; peak intensities were log-transformed before analysis. After exclusion of one contaminated sample and 4 technical failures, the proteomics data consisted of 470 spectra from 138 patients.

### Protein enrichment and identification

To identify the proteins present in the 15.1 kDa and 15.9 kDa peaks, ten µL of a single patient’s serum were applied to Top 12 Abundant Protein Depletion Spin Columns (Thermo Scientific Pierce) according to the manufacturer’s protocol. Five hundred µL of the eluate were concentrated on a 3 kDa molecular weight cut-off column (Amicon) in 25 mM Tris-HCl, pH 8.0. Twenty µL of depleted serum were separated by 1D SDS-PAGE on an 18% Tris-glycine denaturing gel (TGX, Bio-Rad) at 150 V for 70 min and compared against SeeBlue Plus 2 pre-stained protein standard (Life Technologies). The gel was rinsed 5 times with deionized water and stained overnight in See Band staining solution (Gene Bio-Application Ltd.) A band corresponding to 15 to 16 kDa was excised and an in-gel trypsin digest ^13^ was carried out.

Samples were analysed by nanoscale liquid chromatography-electrospray ionization tandem mass spectrometry (LC-MS/MS), using a nanoAcquity UPLC system (Waters MS Technologies, Manchester, UK). Peptide identification was performed using ProteinLynx Global SERVER v3.1 (Waters). 

### Serum haemoglobin concentration

Free haemoglobin levels were assayed by ELISA (Abcam ab157707) according to the manufacturer’s protocol. Samples were analysed in random order, and staff were blinded to the treatment arms. Absorbance was measured at 450 nm on a SpectraMax microplate reader (Molecular Devices).

### Serum lactate dehydrogenase (LDH) activity

Serum LDH activity was assayed by the conversion of lactate to pyruvate, using the absorption of light at 340 nm by the reaction product NADH (Abbott Laboratories, ref. 7D69).

### Statistics

All statistical models were carried out using R software ^14^. To test for associations between SELDI-TOF mass spectrometry peak intensity changes and treatment group, linear regression models were fitted separately for each spectral peak at each follow-up time (6, 12 and 24 months), modelling log(peak intensity change from baseline) as a function of baseline log(peak intensity), treatment group, and the five randomization variables (age, gender, EDSS [Expanded Disability Severity Scale], neuroscience centre, and assessing physician).

To test for associations between peak intensity changes and brain volume changes, for each pair of time points (0–12 months, 0–25 months and 12–25 months) the BBSI was compared with the change in each peak intensity. Linear regression was used to model the log(change in peak intensity) as a function of BBSI (expressed as a percentage of baseline whole-brain volume), adjusted for baseline log(peak intensity), MRI centre, and the five randomization variables.

For both treatment and brain volume analyses, sensitivity analysis was carried out using repeated-measures models including all four time points. Protein peaks whose regression coefficients differed significantly from zero (Wald test) were selected for further analysis. To take into account multiple testing, the p-value for each peak was converted into the False Discovery Rate (FDR: expected proportion of false positives) for that p-value threshold, using the R package fdrtool ^15^. Peaks at FDR ≤ 0.2 were retained for further analysis.

The results of the haemoglobin ELISA and the serum LDH assay were analysed using two-tailed Mann-Whitney tests to test for pairwise differences between the subject groups.

## Results

### Intensity of specific protein peaks was associated with change in brain volume

Expression-difference mapping of all longitudinal serum samples (peak threshold of 12.5%; mass window of 0.3% minimum) resulted in detection of 145 peaks that were differentially expressed within individual subjects over time.

To determine whether changes in protein levels (SELDI-TOF peak intensity) were associated with simvastatin treatment, we ran regression models for the change from baseline in each protein peak intensity vs. treatment status, adjusting for the 5 randomization variables and MRI centre. The 3 follow-up time points (6, 12 and 24 months) were analysed separately. No association remained significant after adjusting for multiple comparisons (the lowest level at which the FDR could be controlled was 0.3).

**Figure 1.**
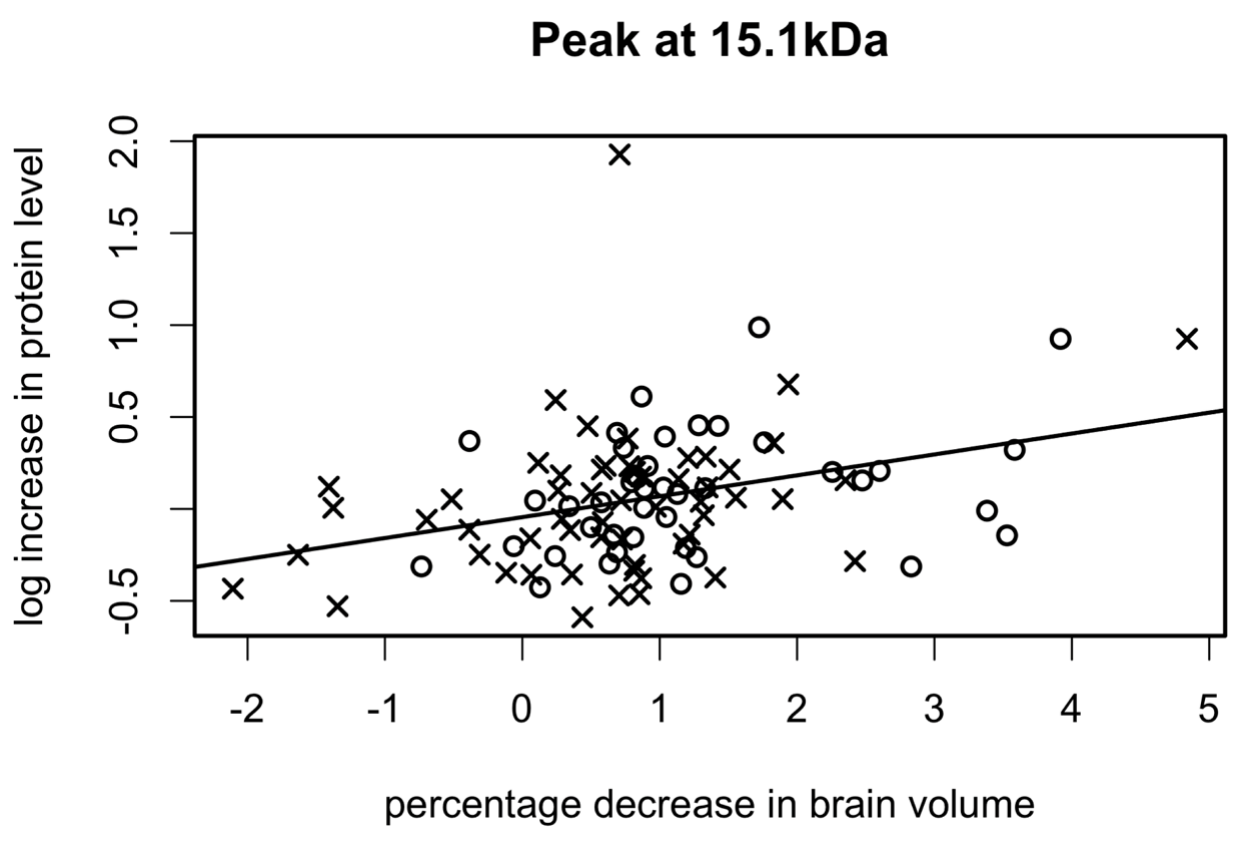
Change in intensity of 15.1 kDa peak correlated with rate of brain atrophy. Normalized log(change in intensity of 15.1 kDa peak between baseline and 2-year follow-up) versus percentage decrease in brain volume between baseline and 2 years. Solid line shows the best fit from the linear regression model (regression coefficient = 0.12, p=0·001). Protein ratios were normalized to all other covariates, using the linear regression model. Crosses represent treated individuals, circles represent untreated individuals. The correlation with the 15.9 kDa peak was closely similar (regression coefficient = 0.12, p=0·001).

We next ran regression models for change in protein intensity v. brain volume loss, for each interval in which the BBSI was measured (0 to 12 months; 12 to 25 months) and over the whole trial period (0 to 25 months). The changes in intensity of peaks at m/z = 25,110 and 25,402 were significantly associated with the change in brain volume between baseline and 12 months (p=0.001, corresponding to FDR = 0.08). The regression coefficients for the association were negative, i.e. an increase in these protein intensities was associated with a smaller decrease in brain volume. The change in intensity of the peaks at m/z = 15,141 and 15,885 between baseline and 25 months was significant in each case (p=0.003 and 0.001 respectively), corresponding to a FDR of 0.2. For these peaks the regression coefficients were positive, i.e. an increase in these peaks was associated with a larger decrease in brain volume ( Figure 1). There were no significant regression coefficients for the 12 to 25 month time period. Repeated-measures models for the whole time period of the trial also identified the peaks at m/z = 15,141 and 15,885 as significant at an FDR of 0.2.

Multiple regression analysis, modelling brain volume change as a function of protein peak intensity, treatment status, and the five randomization variables and MRI centre as covariates confirmed ( Table 1) that simvastatin treatment and the protein peak intensity were independently associated with the rate of brain atrophy.

**Table 1.** Multiple linear regression analysis of the association between change in brain volume and change in the peak intensity of the proteins whose changes were found to be significantly correlated with change in brain volume. Beta = regression coefficient. The model is adjusted for the 5 randomization variables and MRI centre.

	m/z 15885 (0 to 25 months)		m/z 15141 (0 to 25 months)	
	Beta (95% CI)	p	Beta (95% CI)	p
protein	0.83 (0.33, 1.33)	0.001	0.75 (0.29, 1.22)	0.002
treatment	-0·62 (-1.06, -0.19)	0.005	-0.60 (-1.03, -0.17)	0.007
	m/z 25110 (0 to 12 months)		m/z 25402 (0 to 12 months)	
	Beta (95% CI)	p	Beta (95% CI)	p
protein	-0.99 (-1.72, -0.26)	0.008	-0.73 (-1.37, -0.09)	0.025
treatment	-0.26 (-0.56, -0.04)	0.090	-0.27 (-0.58, -0.04)	0.083

The multiple regression model ( Table 1) explained 25% of the observed variation in the rate of brain atrophy over the two-year observation period; the protein peak at 15.1 kDa alone explained 10% of this variation. The regression coefficient of -0.6 for simvastatin treatment indicates a mean difference in brain atrophy rate between treatment groups of -0.6% over the 2-year trial period; this estimate (-0.3%/year) is consistent with the rate of -0.25%/year previously reported in the MS-STAT trial ^1^. The regression coefficient of 0.75 for BBSI v. protein change means that two patients whose protein increases differ by 30% have an expected difference in brain atrophy rate of 0.1% over two years (the patient with higher increase in protein 15.1kDa expects a greater decrease in brain volume).

### Identification of proteins associated with brain atrophy

**Figure 2.**
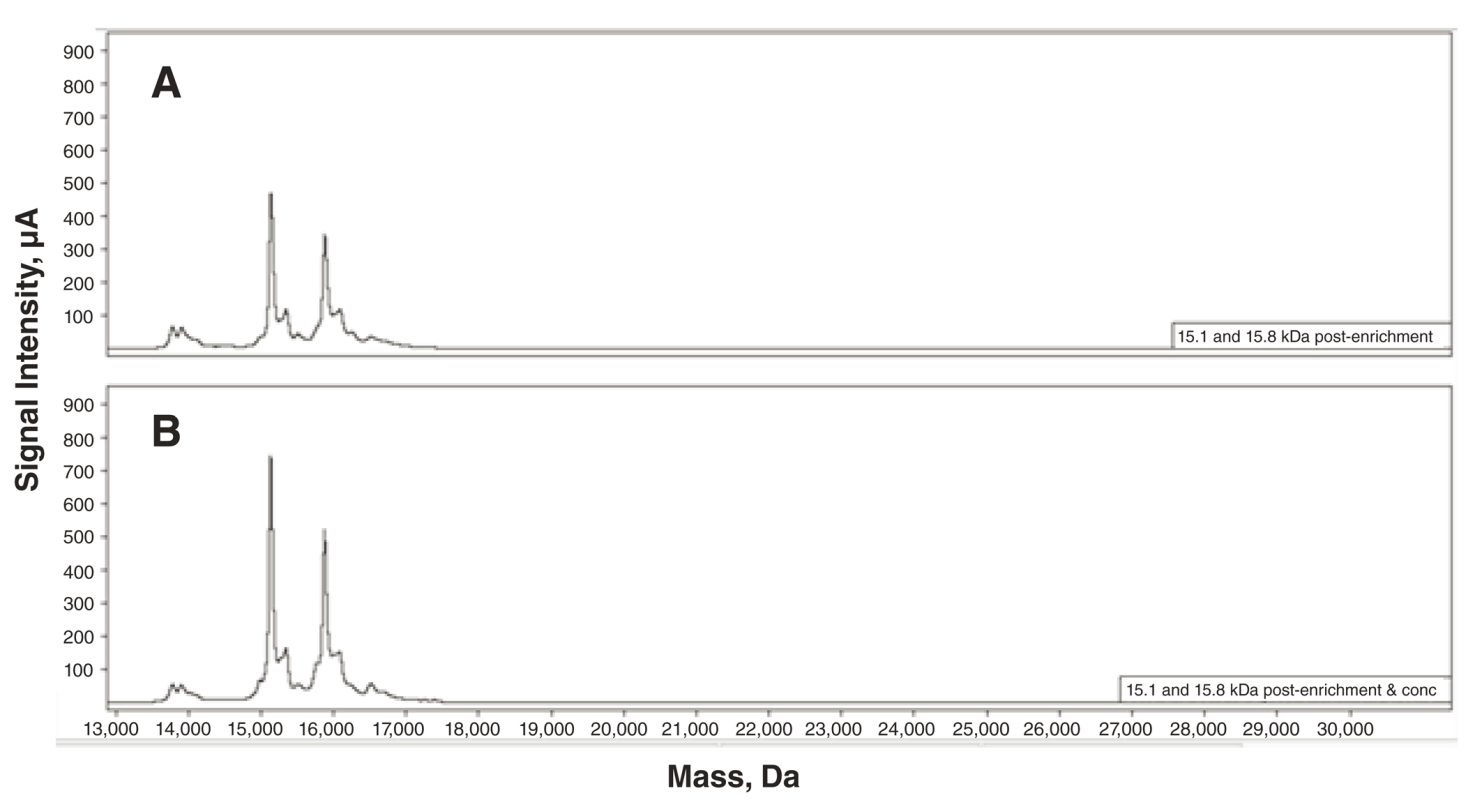
SELDI-TOF mass spectrometry spectra of 15.1 kDa and 15.9 kDa peaks. ( **A.**) Following enrichment on Top 12 Protein Depletion column and ( **B.**) Concentration of eluate on 3 kDa molecular weight cutoff column.

After enrichment, the intensity of the protein peaks at 25.1 kDa and 25.4 kDa remained insufficient to allow their isolation and identification. However, the peaks at 15.1 kDa and 15.9 kDa remained at high intensities and distinct from nearby peaks ( Figure 2). LC-MS/MS identified twenty-six peptide fragments matching human proteins: 15 fragments corresponded to human α-haemoglobin and the remaining 11 fragments corresponded to β-haemoglobin ( Table 2; Supplementary Table 1). Of the remaining 364 sequence matches (after exclusion of bacterial sequences and the common contaminant keratin), the top 360 were partial matches to haemoglobin subunits of other mammalian species.

**Table 2.** Identification of peptide fragments from 15.1 kDa and 15.9 kDa protein peaks, using LC-MS/MS. ^*^oxidation of M(1). ^**^oxidation of M(15).

Peak	m/z	Protein name	Accession no. (UniProt)	PLGS score	Peptide matches
1	15,141	Haemoglobin alpha	P69905	2049	(R)VDPVNFK(L) (R)MFLSFPTTK(T) ^*^ (K)VGGHAAEYGAEALER(M) (R)MFLSFPTTK(T)
2	15,885	Haemoglobin beta	P68871	2579	(R)FFESFGDLSTPDAVMGNPK(V) ^**^ (R)LLVVYPWTQR(F) (K)EFTPPVQAAYQK(V) (R)FFESFGDLSTPDAVMGNPK(V) (K)LHVDPENFR(L)

### ELISA confirms the presence of free serum haemoglobin in MS patients

**Figure 3.**
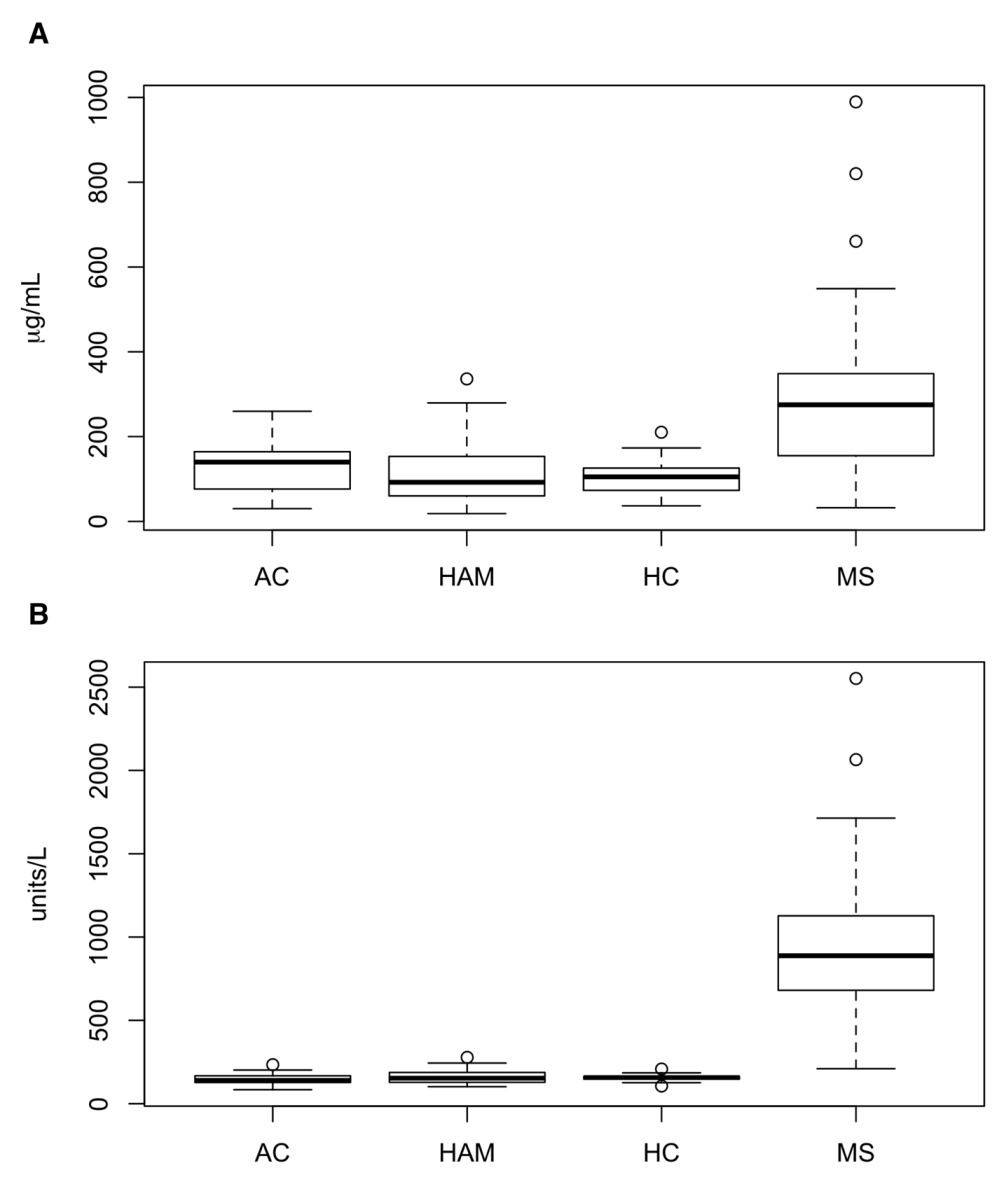
Free serum haemoglobin and lactate dehydrogenase are raised in secondary progressive multiple sclerosis. **A.** Serum haemoglobin concentration measured by ELISA in asymptomatic carriers of HTLV-1 (AC), patients with HTLV-1-associated myelopathy (HAM), uninfected, healthy controls (HC), and patients with secondary progressive MS. The concentration in the MS group was significantly greater than that in the ACs (p = 9.9 × 10 ^-4^), the HAM patients (p = 1.2 × 10 ^-3^), and the HCs (p = 1.6 × 10 ^-4^) (Mann-Whitney test, Bonferroni-corrected). **B**. Serum lactate dehydrogenase (LDH) activity in the same groups of subjects. The LDH activity in the MS group was significantly greater than that in the ACs (p = 2.1 × 10 ^-12^), the HAM patients (p = 2.6 × 10 ^-12^), and the HCs (p = 2.0 × 10 ^-13^) (Mann-Whitney test, Bonferroni-corrected).

We assayed free haemoglobin by ELISA in MS patients (n=20, randomly selected from the study cohort) and in three control groups (n=20 in each group; Materials and Methods). The results ( Figure 3A) showed significantly higher concentrations of free haemoglobin in the serum from MS patients, when compared to each control group (p<0.001 in each comparison; Mann-Whitney). Of the 20 MS patients assayed, 17 had a serum haemoglobin concentration greater than the mean + 2 standard errors of the healthy adult controls. No significant difference was observed between the three control groups. Log [haemoglobin] measured by ELISA was significantly correlated with log(peak intensity)(r = 0.52; p = 0.02; linear regression).

### Abnormally high serum LDH activity in MS patients

The presence of free haemoglobin in the serum in MS patients suggested a degree of intravascular haemolysis in these individuals. To seek corroborative evidence of haemolysis, we assayed the serum LDH activity. The median LDH activity in the patients with MS was significantly greater than that in each of the three control groups ( Figure 3B; p<10 ^-12^ in each case; Mann-Whitney); no significant difference was found between the three control groups, in each of which the LDH was within the normal range.

The mean erythrocyte count and haematocrit in the cohort were within the normal range (see Data availability), and there was no association between these parameters and the rate of brain atrophy. The concentration of free serum haemoglobin observed here (~300 μg/mL; Figure 3A) accounted for only ~0.1% of the total blood haemoglobin, and consequently the mean total blood haemoglobin in the study cohort was also in the normal range.

## Discussion

The characteristic pathological feature of early, active multiple sclerosis lesions is primary demyelination, with partial preservation of axons. But the dominant feature in progressive disease is neurodegeneration, which results in brain atrophy. Factors associated with this neurodegeneration ^5^ include microglial activation, chronic oxidative injury, mitochondrial damage in axons, and iron accumulation. Risk factors of cardiovascular diseases are also associated with lesion burden and brain atrophy in MS ^16^. A strong correlate of the neurodegeneration in MS is abnormal iron deposition in both grey and white matter, especially along veins and venules in cerebral MS plaques ^17, 18^. Iron can potentiate oxidative damage by generating hydroxyl radicals by the Fenton reaction. The extent of iron accumulation, as indicated by T2 signal hypointensity on MRI, is correlated with disease progression, lesion accumulation and cell death of oligodendrocytes ^19– 21^. The extent of iron deposition is greater in SPMS than in relapsing-remitting disease ^19^. In a longitudinal study of 144 patients of whom 62 had relapsing-remitting MS, iron deposition was most marked in early disease ^22^.

The source of this abnormal iron deposited in the central nervous system in MS is unknown. Iron is liberated from damaged oligodendrocytes and myelin ^23^ and accumulates in macrophages and microglia at the margin of active lesions, but it remains unclear whether this is the principal source of the iron that accumulates in the vessel walls and perivascular space. Bamm and Harauz ^24^ suggested that haemoglobin might enter the parenchyma of the central nervous system as a result of either intravascular haemolysis or extravasation of red blood cells ^25, 26^. Both mechanisms could contribute; intravascular haemolysis is more likely to cause a detectable rise in free serum haemoglobin.

The results presented here show that a rise in the concentration of free haemoglobin in the serum was associated with the rate of brain atrophy in this cohort of patients with SPMS. This effect was independent of the beneficial treatment effect of simvastatin, because there was no association between free haemoglobin concentration and simvastatin treatment. Since a successful response to simvastatin treatment was not associated with the free serum haemoglobin concentration, we infer that the change in free serum haemoglobin was not a consequence of brain atrophy but preceded brain atrophy in the causal pathway.

These results suggest the hypothesis that chronic, low-grade intravascular haemolysis releases haemoglobin into the serum, which is then translocated into the CNS parenchyma across the impaired blood-brain barrier and potentiates oxidative damage to oligodendrocytes. Free haemoglobin may itself disrupt the blood-brain barrier ^27^. The toxic effects of free haemoglobin ^28^ can be mediated by intact haemoglobin itself, by haem, or by iron, especially as Fe ^2+^ ^24^. Haemoglobin is degraded by haem oxygenase-1 (HO-1), producing biliverdin and Fe ^2+^ ions. HO-1 is upregulated in glia by oxidative stress, and HO-1 is expressed in oligodendrocytes in actively demyelinating areas in MS, but not in two other CNS diseases, human acute disseminated leukoencephalomyelitis (ADEM) or murine experimental allergic encephalomyelitis (EAE) ^29^. Stahnke *et al.* ^29^ proposed that the role of stress-induced HO-1 is protective initially, whereas chronic upregulation might cause oligodendrocyte death. Altinoz *et al.* ^30^ reviewed evidence from genetic surveys and *in vitro* studies and proposed that haemoglobins could contribute to the pathogenesis of MS in both the “inside-out” and “outside-in” models ^5^. Ozcan *et al.* recently reported a negative correlation between the serum level of the minor haemoglobin A2 (HbA2) and disease severity in MS ^31^; HbA2 and its breakdown product hemichrome A2 bind the erythrocyte membrane with higher affinity than major haemoglobin, and may diminish erythrocyte fragility ^32^.

The observation ( Figure 3B) of abnormally high serum LDH activity is consistent with the presence of haemolysis in these patients. LDH is present in all cell types, and serum LDH is raised in many inflammatory conditions; however, erythrocytes are particularly rich in LDH, and serum LDH is a sensitive marker of haemolysis. The notion that chronic intravascular haemolysis might serve as a source of the iron deposited in MS is also consistent with earlier reports of abnormal size ^33^ and fragility ^34– 37^ of erythrocytes in the disease. Erythrocytes from patients with MS, especially those with active disease, are abnormally susceptible to lysis by both mechanical stress ^36^ and osmotic stress ^34, 35, 37^. The causes of this erythrocyte fragility remain to be identified. Possible artefactual causes of haemolysis, such as venepuncture, cannot explain the significant association observed here between brain atrophy and free serum haemoglobin.

If intravascular haemolysis indeed occurs in SPMS, the rate of red cell destruction is insufficient to reduce the total blood haemoglobin, which remained within normal limits in this cohort. Neurodegeneration is not a feature of other chronic haemolytic conditions, such as spherocytosis or elliptocytosis; however, in these conditions the blood-brain barrier is intact, and most erythrocyte destruction occurs in the spleen, where efficient phagocytosis may prevent the release of the toxic breakdown products into the circulation.

Polymorphisms in genes encoding iron-binding and iron-transporting proteins are associated with disability, disease severity and early progression in MS ^38^. Rithidech *et al*. ^39^ used 2D electrophoresis to identify plasma biomarkers in paediatric MS: the haem-binding protein haemopexin was 1 of 12 proteins found to be upregulated in 9 MS patients. Robotti *et al*. ^40^ identified an alteration in the ratio of isoforms of haptoglobin (which bind free haemoglobin) in MS. The frequency of polymorphic variants of haptoglobin varies between geographical regions, and genetic epidemiological studies are needed to test whether particular haptoglobin alleles are associated with disease severity in MS ^25^.

These results do not suggest that free serum haemoglobin concentration is useful in the differential diagnosis of neurological disease; rather, they identify a potential contributor to the pathogenesis of neurodegeneration in progressive multiple sclerosis. It is possible that other, lower-abundance markers are also correlated with brain atrophy rate. Other markers could be sought both by fractionating diluted serum and by using other surfaces in the SELDI protocol in order to select subsets of proteins with different profiles of hydrophobicity and electric charge.

Previous studies of serum iron have shown normal concentrations in the serum in patients with MS ^41^; however, standard assays of free serum iron do not detect iron that is sequestered in haemoglobin. Iron chelation has been proposed as a therapy to approach to reduce neurodegeneration in MS. However, if an important source of iron is free serum haemoglobin, standard iron-chelating agents such as desferrioxamine may be ineffective, again because the iron is sequestered in haemoglobin ^42^. Scavengers of haemoglobin and haemin ^43^ might be more effective.

## Data availability

Links to each respective dataset are given below. The data on SELDI-TOF peaks are derived from the BioRad software package ProteinChip Data Manager.

Zenodo: Raw data for SELDI-TOF low range from article: Free serum haemoglobin is associated with brain atrophy in secondary progressive multiple sclerosis, http://doi.org/10.5281/zenodo.160737 ^44^

Zenodo: Raw data for SELDI-TOF high range from article: Free serum haemoglobin is associated with brain atrophy in secondary progressive multiple sclerosis, http://doi.org/10.5281/zenodo.160743 ^45^

Zenodo: Nano LC MS-MS peptide matches from article: Free serum haemoglobin is associated with brain atrophy in secondary progressive multiple sclerosis, http://doi.org/10.5281/zenodo.160744 ^46^

Zenodo: Raw data for RBC, Hb and haematocrit from article: Free serum haemoglobin is associated with brain atrophy in secondary progressive multiple sclerosis, http://doi.org/10.5281/zenodo.161532 ^47^

## Supplementary material

**Supplementary Table 1:** Protein peaks associated with brain atrophy: identification by liquid chromatography-electrospray ionization tandem mass spectrometry.

Relative molecular mass, protein score and identity of the genes with sequence matches to peptide fragments from the protein peaks that were significantly correlated with the rate of brain atrophy. The common contaminant keratin and partial matches to contaminating bacterial sequences were excluded.
