# Supplementary material for: Free serum haemoglobin is associated with brain atrophy in secondary progressive multiple sclerosis
Source: Wellcome Open Res. 2016 Dec 23;1:10. Originally published 2016 Nov 15. [Version 2] doi: 10.12688/wellcomeopenres.9967.2 (PMC5159626; doi:10.12688/wellcomeopenres.9967.2)
Supplement: Supplementary file 1 [file wellcomeopenres-1-11315-s0000.tgz › 10399af0-5b56-48a1-98cf-033688a1b9bb.pdf]

Protein peaks associated with brain atrophy: identification by liquid chromatography-electrospray ionization tandem mass spectrometry

protein key	protein Entry	protein Accession	protein Description	protein score	protein avg Mass	protein Matched Products	protein matched peptides	protein digest Peps	protein seq Cover (%)	protein Matched Peptide Intensity Sum	protein top3 Matched Peptide Intensity Sum	protein Matched Product Intensity Sum
163295	HBB_PANTR	P68873	Hemoglobin subunit beta OS=Pan troglodytes GN=HBB PE=1 SV=2	8374.811	16114.45	121	7	13	47.62	831533	0	219414
163293	HBB_PANPA	P68872	Hemoglobin subunit beta OS=Pan paniscus GN=HBB PE=1 SV=2	8374.811	16114.45	121	7	13	47.62	831533	0	219414
**163232**	**HBB_HUMAN**	**P68871**	**Hemoglobin subunit beta OS=Homo sapiens GN=HBB PE=1 SV=2**	**8374.811**	**16114.45**	**121**	**7**	**13**	**47.62**	**831533**	**584051**	**219414**
163224	HBB_GORGO	P02024	Hemoglobin subunit beta OS=Gorilla gorilla gorilla GN=HBB PE=1 SV=2	8019.335	16086.436	114	6	13	41.5	818936	0	216132
163234	HBB_HYLLA	P02025	Hemoglobin subunit beta OS=Hylobates lar GN=HBB PE=1 SV=1	7075.644	16041.291	103	6	12	39.73	524552	0	154517
163314	HBB_PROLO	P18989	Hemoglobin subunit beta OS=Procyon lotor GN=HBB PE=1 SV=1	4736.479	16123.434	66	5	12	34.93	603497	0	128247
163312	HBB_PROCR	P23020	Hemoglobin subunit beta OS=Proteles cristata GN=HBB PE=1 SV=1	4381.005	16103.313	59	4	10	28.77	590900	0	124965
163201	HBB_CROCR	P18986	Hemoglobin subunit beta OS=Crocuta crocuta GN=HBB PE=1 SV=1	4381.005	16133.339	59	4	10	28.77	590900	0	124965
163397	HBD_PONPY	P61775	Hemoglobin subunit delta OS=Pongo pygmaeus GN=HBD PE=2 SV=2	3437.313	16139.46	48	4	13	26.53	296516	0	63350
163395	HBD_PANTR	P61772	Hemoglobin subunit delta OS=Pan troglodytes GN=HBD PE=1 SV=2	3437.313	16139.46	48	4	13	26.53	296516	0	63350
163392	HBD_HYLLA	P61774	Hemoglobin subunit delta OS=Hylobates lar GN=HBD PE=1 SV=2	3437.313	16139.46	48	4	13	26.53	296516	0	63350
163391	HBD_HUMAN	P02042	Hemoglobin subunit delta OS=Homo sapiens GN=HBD PE=1 SV=2	3437.313	16171.526	48	4	13	26.53	296516	0	63350
163389	HBD_GORGO	P61773	Hemoglobin subunit delta OS=Gorilla gorilla gorilla GN=HBD PE=1 SV=2	3437.313	16139.46	48	4	13	26.53	296516	0	63350
163384	HBD_COLPO	P19886	Hemoglobin subunit delta OS=Colobus polykomos GN=HBD PE=2 SV=2	3437.313	16050.406	48	4	13	26.53	296516	0	63350
163352	HBB_TAMHU	B3EWD8	Hemoglobin subunit beta OS=Tamiasciurus hudsonicus PE=1 SV=1	3437.313	15913.167	48	4	12	26.71	296516	0	63350
163341	HBB_SPEBE	B3EWD0	Hemoglobin subunit beta OS=Spermophilus beecheyi PE=1 SV=1	3437.313	15883.102	48	4	12	26.71	296516	0	63350
163336	HBB_SEMEN	P02032	Hemoglobin subunit beta OS=Semnopithecus entellus GN=HBB PE=1 SV=1	3437.313	16011.265	48	4	12	26.71	296516	0	63350
163331	HBB_SAISC	P02036	Hemoglobin subunit beta OS=Saimiri sciureus GN=HBB PE=1 SV=2	3437.313	16159.45	48	4	13	26.53	296516	0	63350
163330	HBB_SAGOE	P68055	Hemoglobin subunit beta OS=Saguinus oedipus GN=HBB PE=1 SV=1	3437.313	16088.304	48	4	12	26.71	296516	0	63350
163329	HBB_SAGNI	P68054	Hemoglobin subunit beta OS=Saguinus nigricollis GN=HBB PE=1 SV=1	3437.313	16088.304	48	4	12	26.71	296516	0	63350

163328	HBB_SAGMY	P02038	Hemoglobin subunit beta OS=Saguinus mystax GN=HBB PE=1 SV=1	3437.313	16074.278	48	4	12	26.71	296516	0	63350
163327	HBB_SAGFU	P02039	Hemoglobin subunit beta OS=Saguinus fuscicollis GN=HBB PE=1 SV=1	3437.313	16030.268	48	4	12	26.71	296516	0	63350
163325	HBB_RHIUN	P09907	Hemoglobin subunit beta OS=Rhinoceros unicornis GN=HBB PE=1 SV=1	3437.313	15915.073	48	4	10	26.71	296516	0	63350
163320	HBB_RABIT	P02057	Hemoglobin subunit beta-1/2 OS=Oryctolagus cuniculus GN=HBB1 PE=1 SV=2	3437.313	16190.559	48	4	13	26.53	296516	0	63350
163319	HBB_PTEPO	P14392	Hemoglobin subunit beta OS=Pteropus poliocephalus GN=HBB PE=1 SV=1	3437.313	16063.385	48	4	12	26.71	296516	0	63350
163318	HBB_PTEGI	D0VX08	Hemoglobin subunit beta OS=Pteropus giganteus GN=HBB PE=1 SV=1	3437.313	16053.346	48	4	12	26.71	296516	0	63350
163316	HBB_PTEAL	P14391	Hemoglobin subunit beta OS=Pteropus alecto GN=HBB PE=1 SV=1	3437.313	16053.346	48	4	12	26.71	296516	0	63350
163308	HBB_PITPI	Q6WN27	Hemoglobin subunit beta OS=Pithecia pithecia GN=HBB PE=2 SV=3	3437.313	16159.45	48	4	13	26.53	296516	0	63350
163306	HBB_PIG	P02067	Hemoglobin subunit beta OS=Sus scrofa GN=HBB PE=1 SV=3	3437.313	16223.595	48	4	13	26.53	296516	0	63350
163281	HBB_NYCCO	P02049	Hemoglobin subunit beta OS=Nycticebus coucang GN=HBB PE=1 SV=1	3437.313	15958.212	48	4	12	26.71	296516	0	63350
163278	HBB_NASNA	P26916	Hemoglobin subunit beta OS=Nasua nasua GN=HBB PE=1 SV=1	3437.313	16193.485	48	4	12	26.71	296516	0	63350
163250	HBB_LYNLY	P41328	Hemoglobin subunit beta OS=Lynx lynx GN=HBB PE=1 SV=1	3437.313	16041.251	48	4	10	26.71	296516	0	63350
163247	HBB_LORTA	P02048	Hemoglobin subunit beta OS=Loris tardigradus GN=HBB PE=1 SV=1	3437.313	15958.212	48	4	12	26.71	296516	0	63350
163235	HBB_LAGLA	Q6WN25	Hemoglobin subunit beta OS=Lagothrix lagotricha GN=HBB PE=2 SV=3	3437.313	16132.425	48	4	13	26.53	296516	0	63350
163231	HBB_HORSE	P02062	Hemoglobin subunit beta OS=Equus caballus GN=HBB PE=1 SV=1	3437.313	16066.311	48	4	12	26.71	296516	0	63350
163221	HBB_FELCA	P07412	Hemoglobin subunit beta-A/B OS=Felis catus GN=HBB PE=1 SV=1	3437.313	16042.235	48	4	10	26.71	296516	0	63350
163217	HBB_EQUZE	P67824	Hemoglobin subunit beta OS=Equus zebra GN=HBB PE=1 SV=1	3437.313	16137.39	48	4	12	26.71	296516	0	63350
163216	HBB_EQUHE	P67823	Hemoglobin subunit beta OS=Equus hemionus kulan GN=HBB PE=1 SV=1	3437.313	16137.39	48	4	12	26.71	296516	0	63350
163204	HBB_CTEGU	P20855	Hemoglobin subunit beta OS=Ctenodactylus gundi GN=HBB PE=1 SV=1	3437.313	15904.184	48	4	10	26.71	296516	0	63350
163185	HBB_CERSI	P02066	Hemoglobin subunit beta OS=Ceratotherium simum GN=HBB PE=1 SV=1	3437.313	16025.386	48	4	12	26.71	296516	0	63350
163182	HBB_CEBAP	P67821	Hemoglobin subunit beta OS=Cebus apella GN=HBB PE=1 SV=3	3437.313	16249.529	48	4	13	26.53	296516	0	63350
163173	HBB_CALTO	Q6WN20	Hemoglobin subunit beta OS=Callicebus torquatus GN=HBB PE=2 SV=3	3437.313	15950.246	48	4	12	26.53	296516	0	63350
163172	HBB_CALJA	Q6WN28	Hemoglobin subunit beta OS=Callithrix jacchus GN=HBB PE=2 SV=3	3437.313	16199.515	48	4	13	26.53	296516	0	63350
163171	HBB_CALGO	Q6WN21	Hemoglobin subunit beta OS=Callimico goeldii GN=HBB PE=2 SV=3	3437.313	16208.477	48	4	13	26.53	296516	0	63350

163170	HBB_CALAR	P18985	Hemoglobin subunit beta OS=Callithrix argentata GN=HBB PE=1 SV=1	3437.313	16068.317	48	4	12	26.71	296516	0	63350
163158	HBB_BALAC	P18984	Hemoglobin subunit beta OS=Balaenoptera acutorostrata GN=HBB PE=1 SV=1	3437.313	16119.539	48	4	12	26.71	296516	0	63350
163156	HBB_ATEPA	Q6WN22	Hemoglobin subunit beta OS=Ateles paniscus GN=HBB PE=1 SV=3	3437.313	16162.451	48	4	13	26.53	296516	0	63350
163155	HBB_ATEGE	P68232	Hemoglobin subunit beta OS=Ateles geoffroyi GN=HBB PE=1 SV=2	3437.313	16160.479	48	4	13	26.53	296516	0	63350
163154	HBB_ATEBE	P68234	Hemoglobin subunit beta OS=Ateles belzebuth GN=HBB PE=1 SV=2	3437.313	16160.479	48	4	13	26.53	296516	0	63350
163147	HBB_AOTTR	P02035	Hemoglobin subunit beta OS=Aotus trivirgatus GN=HBB PE=1 SV=1	3437.313	16014.225	48	4	12	26.71	296516	0	63350
163139	HBB_ALOBE	Q6WN29	Hemoglobin subunit beta OS=Alouatta belzebul GN=HBB PE=2 SV=3	3437.313	16173.477	48	4	13	26.53	296516	0	63350
163064	HBB1_TAPTE	P02064	Hemoglobin subunit beta-1 OS=Tapirus terrestris GN=HBB1 PE=1 SV=1	3437.313	16031.343	48	4	11	26.71	296516	0	63350
163404	HBD_TRIMA	Q45XI8	Hemoglobin subunit delta OS=Trichechus manatus GN=HBD PE=2 SV=3	3425.654	16426.821	46	3	14	21.09	295628	0	62388
163364	HBB_TRIIN	P07415	Hemoglobin subunit beta OS=Trichechus inunguis GN=HBB PE=1 SV=1	3425.654	16295.623	46	3	13	21.23	295628	0	62388
163348	HBB_SUNMU	P02060	Hemoglobin subunit beta OS=Suncus murinus GN=HBB PE=1 SV=1	3425.654	15850.142	46	3	12	21.23	295628	0	62388
163337	HBB_SHEEP	P02075	Hemoglobin subunit beta OS=Ovis aries GN=HBB PE=1 SV=2	3425.654	16131.474	46	3	11	21.38	295628	0	62388
163335	HBB_SCICA	B3EWD2	Hemoglobin subunit beta OS=Sciurus carolinensis PE=1 SV=1	3425.654	15972.233	46	3	12	21.23	295628	0	62388
163305	HBB_PHYCD	P09905	Hemoglobin subunit beta-1/2 OS=Physeter catodon GN=HBB PE=1 SV=1	3425.654	16241.669	46	3	12	21.23	295628	0	62388
163242	HBB_LEPEU	P08535	Hemoglobin subunit beta OS=Lepus europaeus GN=HBB PE=1 SV=2	3425.654	16120.508	46	3	13	21.09	295628	0	62388
163376	HBD_AILME	Q5XLE5	Hemoglobin subunit delta OS=Ailuropoda melanoleuca GN=HBD PE=2 SV=3	3081.838	16296.67	41	3	13	20.41	283919	0	60068
163371	HBB_URSTH	P68012	Hemoglobin subunit beta OS=Ursus thibetanus GN=HBB PE=1 SV=1	3081.838	16125.407	41	3	12	20.55	283919	0	60068
163370	HBB_URSMA	P68011	Hemoglobin subunit beta OS=Ursus maritimus GN=HBB PE=1 SV=1	3081.838	16125.407	41	3	12	20.55	283919	0	60068
163358	HBB_THEGE	P02029	Hemoglobin subunit beta OS=Theropithecus gelada GN=HBB PE=1 SV=1	3081.838	16041.291	41	3	12	20.55	283919	0	60068
163317	HBB_PTEBR	P68052	Hemoglobin subunit beta OS=Pteronura brasiliensis GN=HBB PE=1 SV=1	3081.838	16050.339	41	3	12	20.55	283919	0	60068
163307	HBB_PILBA	P02033	Hemoglobin subunit beta OS=Piliocolobus badius GN=HBB PE=1 SV=1	3081.838	15986.212	41	3	12	20.55	283919	0	60068
163304	HBB_PHOVI	P09909	Hemoglobin subunit beta OS=Phoca vitulina GN=HBB PE=1 SV=1	3081.838	16040.301	41	3	12	20.55	283919	0	60068
163297	HBB_PAPCY	P02030	Hemoglobin subunit beta OS=Papio cynocephalus GN=HBB PE=1 SV=1	3081.838	16011.265	41	3	12	20.55	283919	0	60068
163296	HBB_PAPAN	Q9TSP1	Hemoglobin subunit beta OS=Papio anubis GN=HBB PE=2 SV=2	3081.838	16142.463	41	3	13	20.41	283919	0	60068

163282	HBB_ODORO	P68046	Hemoglobin subunit beta OS=Odobenus rosmarus divergens GN=HBB PE=1 SV=1	3081.838	16036.312	41	3	12	20.55	283919	0	60068
163267	HBB_MELME	P02055	Hemoglobin subunit beta OS=Meles meles GN=HBB PE=1 SV=1	3081.838	16126.391	41	3	12	20.55	283919	0	60068
163266	HBB_MELCA	P15449	Hemoglobin subunit beta OS=Mellivora capensis GN=HBB PE=1 SV=1	3081.838	16064.366	41	3	12	20.55	283919	0	60068
163262	HBB_MARFO	P68053	Hemoglobin subunit beta OS=Martes foina GN=HBB PE=1 SV=1	3081.838	16050.339	41	3	12	20.55	283919	0	60068
163261	HBB_MANSP	P08259	Hemoglobin subunit beta OS=Mandrillus sphinx GN=HBB PE=1 SV=1	3081.838	16072.302	41	3	12	20.55	283919	0	60068
163260	HBB_MACSP	P68224	Hemoglobin subunit beta OS=Macaca speciosa GN=HBB PE=1 SV=2	3081.838	16230.526	41	3	13	20.41	283919	0	60068
163258	HBB_MACNE	P68225	Hemoglobin subunit beta OS=Macaca nemestrina GN=HBB PE=1 SV=2	3081.838	16230.526	41	3	13	20.41	283919	0	60068
163254	HBB_MACFU	P68222	Hemoglobin subunit beta OS=Macaca fuscata fuscata GN=HBB PE=1 SV=2	3081.838	16230.526	41	3	13	20.41	283919	0	60068
163253	HBB_MACFA	P68223	Hemoglobin subunit beta OS=Macaca fascicularis GN=HBB PE=1 SV=2	3081.838	16230.526	41	3	13	20.41	283919	0	60068
163249	HBB_LUTLU	P10893	Hemoglobin subunit beta OS=Lutra lutra GN=HBB PE=1 SV=1	3081.838	16066.339	41	3	12	20.55	283919	0	60068
163244	HBB_LEPWE	P15166	Hemoglobin subunit beta OS=Leptonychotes weddelli GN=HBB PE=1 SV=1	3081.838	16109.364	41	3	11	20.55	283919	0	60068
163228	HBB_HELMA	P68013	Hemoglobin subunit beta OS=Helarctos malayanus GN=HBB PE=1 SV=1	3081.838	16125.407	41	3	12	20.55	283919	0	60068
163220	HBB_EULFU	P02053	Hemoglobin subunit beta OS=Eulemur fulvus fulvus GN=HBB PE=1 SV=2	3081.838	15959.26	41	3	9	20.41	283919	0	60068
163198	HBB_COLPO	P19885	Hemoglobin subunit beta OS=Colobus polykomos GN=HBB PE=2 SV=2	3081.838	16100.423	41	3	13	20.41	283919	0	60068
163190	HBB_CHLAE	P02028	Hemoglobin subunit beta OS=Chlorocebus aethiops GN=HBB PE=1 SV=1	3081.838	16043.304	41	3	12	20.55	283919	0	60068
163184	HBB_CERAT	P02031	Hemoglobin subunit beta OS=Cercocebus atys GN=HBB PE=1 SV=1	3081.838	16111.382	41	3	12	20.55	283919	0	60068
163152	HBB_ARCGA	P68047	Hemoglobin subunit beta OS=Arctocephalus galapagoensis GN=HBB PE=1 SV=1	3081.838	16036.312	41	3	12	20.55	283919	0	60068
163146	HBB_AOTAZ	Q6WN26	Hemoglobin subunit beta OS=Aotus azarae GN=HBB PE=2 SV=3	3081.838	16030.335	41	3	12	20.41	283919	0	60068
163135	HBB_AILME	P18983	Hemoglobin subunit beta OS=Ailuropoda melanoleuca GN=HBB PE=1 SV=2	3081.838	16268.616	41	3	13	20.41	283919	0	60068
163134	HBB_AILFU	P18982	Hemoglobin subunit beta OS=Ailurus fulgens GN=HBB PE=1 SV=1	3081.838	16066.339	41	3	12	20.55	283919	0	60068
163387	HBD_DUGDU	Q45XI7	Hemoglobin subunit delta OS=Dugong dugon GN=HBD PE=2 SV=3	3070.179	16193.532	39	2	13	14.97	283031	0	59106
163342	HBB_SPECI	P09421	Hemoglobin subunit beta OS=Spermophilus citellus GN=HBB PE=1 SV=1	3070.179	15817.087	39	2	12	15.07	283031	0	59106
163276	HBB_MUSPU	P68045	Hemoglobin subunit beta OS=Mustela putorius GN=HBB PE=1 SV=1	3070.179	16110.392	39	2	12	15.07	283031	0	59106
163275	HBB_MUSPF	P68044	Hemoglobin subunit beta OS=Mustela putorius furo GN=HBB PE=1 SV=1	3070.179	16110.392	39	2	12	15.07	283031	0	59106

163269	HBB_MESAU	P02094	Hemoglobin subunit beta OS=Mesocricetus auratus GN=HBB PE=1 SV=2	3070.179	16049.412	39	2	13	14.97	283031	0	59106
163263	HBB_MARMA	P08853	Hemoglobin subunit beta OS=Marmota marmota marmota GN=HBB PE=1 SV=1	3070.179	15794.049	39	2	12	15.07	283031	0	59106
163209	HBB_DASNO	P02087	Hemoglobin subunit beta OS=Dasypus novemcinctus GN=HBB PE=1 SV=3	3070.179	16524.961	39	2	13	14.97	283031	0	59106
162844	HBA_BOVIN	P01966	Hemoglobin subunit alpha OS=Bos taurus GN=HBA PE=1 SV=2	2598.529	15184.393	80	5	10	23.24	956689	0	158009
162841	HBA_BISBO	P09423	Hemoglobin subunit alpha-I/II OS=Bison bonasus PE=1 SV=2	2598.529	15139.355	80	5	10	23.24	956689	0	158009
162721	HBA2_BOSMU	P01968	Hemoglobin subunit alpha-2 OS=Bos mutus grunniens PE=1 SV=1	2598.529	14981.131	80	5	9	23.4	956689	0	158009
162687	HBA1_BOSMU	P01967	Hemoglobin subunit alpha-1 OS=Bos mutus grunniens PE=1 SV=1	2598.529	15044.184	80	5	9	23.4	956689	560326	158009
163030	HBA_UROTO	P07403	Hemoglobin subunit alpha OS=Urocitellus townsendii GN=HBA PE=1 SV=1	2476.57	15222.276	79	5	9	17.73	1153082	0	193037
163029	HBA_UROPR	P11750	Hemoglobin subunit alpha OS=Urocitellus parryii GN=HBA PE=1 SV=2	2476.57	15105.179	79	5	9	17.73	1153082	0	193037
163018	HBA_TARBA	P01940	Hemoglobin subunit alpha OS=Tarsius bancanus GN=HBA PE=1 SV=1	2476.57	15302.383	79	5	8	17.73	1153082	0	193037
163016	HBA_TAPGE	P28780	Hemoglobin subunit alpha OS=Taphozous georgianus GN=HBA PE=1 SV=1	2476.57	15169.241	79	5	8	17.73	1153082	0	193037
163014	HBA_TAMMR	B3EWC7	Hemoglobin subunit alpha OS=Tamias merriami PE=1 SV=1	2476.57	15119.206	79	5	9	17.73	1153082	0	193037
163005	HBA_SPECI	P09420	Hemoglobin subunit alpha OS=Spermophilus citellus GN=HBA PE=1 SV=1	2476.57	15308.366	79	5	11	17.73	1153082	0	193037
163004	HBA_SPEBE	B3EWC9	Hemoglobin subunit alpha OS=Spermophilus beecheyi PE=1 SV=1	2476.57	15081.197	79	5	9	17.73	1153082	0	193037
163001	HBA_SEMEN	P01924	Hemoglobin subunit alpha OS=Semnopithecus entellus GN=HBA PE=1 SV=1	2476.57	15179.369	79	5	8	17.73	1153082	0	193037
162998	HBA_SAGOE	P67818	Hemoglobin subunit alpha OS=Saguinus oedipus GN=HBA PE=1 SV=1	2476.57	15152.259	79	5	8	17.73	1153082	0	193037
162997	HBA_SAGMY	Q7M3B6	Hemoglobin subunit alpha OS=Saguinus mystax GN=HBA PE=1 SV=1	2476.57	15128.278	79	5	8	17.73	1153082	0	193037
162996	HBA_SAGFU	P01929	Hemoglobin subunit alpha OS=Saguinus fuscicollis GN=HBA PE=1 SV=1	2476.57	15113.306	79	5	8	17.73	1153082	0	193037
162980	HBA_PONPY	P06635	Hemoglobin subunit alpha OS=Pongo pygmaeus GN=HBA1 PE=2 SV=2	2476.57	15389.62	79	5	10	17.61	1153082	0	193037
162978	HBA_PIPAB	Q862A7	Hemoglobin subunit alpha OS=Pipistrellus abramus GN=HBA PE=2 SV=3	2476.57	15571.751	79	5	9	17.48	1153082	0	193037
162977	HBA_PILBA	P01930	Hemoglobin subunit alpha OS=Piliocolobus badius GN=HBA PE=1 SV=2	2476.57	15285.514	79	5	9	17.61	1153082	0	193037
162964	HBA_PANTR	P69907	Hemoglobin subunit alpha OS=Pan troglodytes GN=HBA1 PE=1 SV=2	2476.57	15315.584	79	5	9	17.61	1153082	0	193037
162961	HBA_PANPA	P69906	Hemoglobin subunit alpha OS=Pan paniscus GN=HBA1 PE=1 SV=2	2476.57	15315.584	79	5	9	17.61	1153082	0	193037
162950	HBA_NYCCO	P01937	Hemoglobin subunit alpha OS=Nycticebus coucang GN=HBA PE=1 SV=1	2476.57	15361.448	79	5	8	17.73	1153082	0	193037

162948	HBA_MYOVE	P11757	Hemoglobin subunit alpha OS=Myotis velifer GN=HBA PE=1 SV=1	2476.57	15255.451	79	5	8	17.73	1153082	0	193037
162935	HBA_MARMA	P08852	Hemoglobin subunit alpha OS=Marmota marmota marmota GN=HBA PE=1 SV=1	2476.57	15133.232	79	5	9	17.73	1153082	0	193037
162933	HBA_MANSP	P08258	Hemoglobin subunit alpha-1/2 OS=Mandrillus sphinx PE=1 SV=1	2476.57	15354.64	79	5	10	17.73	1153082	0	193037
162932	HBA_MACSP	P07402	Hemoglobin subunit alpha-1/2 OS=Macaca speciosa PE=1 SV=1	2476.57	15236.461	79	5	8	17.73	1153082	0	193037
162931	HBA_MACSI	P21768	Hemoglobin subunit alpha-1/2 OS=Macaca sinica PE=1 SV=1	2476.57	15213.424	79	5	9	17.73	1153082	0	193037
162930	HBA_MACNE	P19002	Hemoglobin subunit alpha-1/2/3 OS=Macaca nemestrina PE=1 SV=1	2476.57	15197.424	79	5	8	17.73	1153082	0	193037
162929	HBA_MACMU	P63108	Hemoglobin subunit alpha OS=Macaca mulatta GN=HBA PE=1 SV=2	2476.57	15272.559	79	5	9	17.61	1153082	0	193037
162927	HBA_MACGG	P24659	Hemoglobin subunit alpha-1/2 OS=Macroderma gigas PE=1 SV=1	2476.57	15068.112	79	5	8	17.73	1153082	0	193037
162926	HBA_MACFU	P63107	Hemoglobin subunit alpha OS=Macaca fuscata fuscata GN=HBA PE=1 SV=2	2476.57	15272.559	79	5	9	17.61	1153082	0	193037
162925	HBA_MACFA	P21767	Hemoglobin subunit alpha-A/Q/R/T OS=Macaca fascicularis PE=1 SV=1	2476.57	15169.414	79	5	8	17.73	1153082	0	193037
162922	HBA_MACAS	P21766	Hemoglobin subunit alpha-1/2/3 OS=Macaca assamensis PE=1 SV=1	2476.57	15178.424	79	5	8	17.73	1153082	0	193037
162917	HBA_LORTA	P01938	Hemoglobin subunit alpha OS=Loris tardigradus GN=HBA PE=1 SV=2	2476.57	15362.476	79	5	8	17.73	1153082	0	193037
162908	HBA_HUMAN	P69905	Hemoglobin subunit alpha OS=Homo sapiens GN=HBA1 PE=1 SV=2	2476.57	15315.584	79	5	9	17.61	1153082	0	193037
162901	HBA_GORGO	P01923	Hemoglobin subunit alpha OS=Gorilla gorilla gorilla GN=HBA PE=1 SV=1	2476.57	15170.358	79	5	8	17.73	1153082	0	193037
162898	HBA_EULFU	P01936	Hemoglobin subunit alpha OS=Eulemur fulvus fulvus GN=HBA PE=1 SV=1	2476.57	15196.269	79	5	8	17.73	1153082	0	193037
162880	HBA_CYNSP	P11753	Hemoglobin subunit alpha OS=Cynopterus sphinx GN=HBA PE=1 SV=1	2476.57	15227.337	79	5	8	17.73	1153082	0	193037
162865	HBA_CHLAE	P01926	Hemoglobin subunit alpha OS=Chlorocebus aethiops GN=HBA PE=1 SV=2	2476.57	15295.596	79	5	9	17.61	1153082	0	193037
162862	HBA_CHAMP	Q7M2Y4	Hemoglobin subunit alpha OS=Chalinolobus morio GN=HBA PE=1 SV=1	2476.57	15185.212	79	5	8	17.73	1153082	0	193037
162859	HBA_CEBCA	P07421	Hemoglobin subunit alpha OS=Cebus capucinus GN=HBA PE=1 SV=1	2476.57	15182.286	79	5	8	17.73	1153082	0	193037
162858	HBA_CEBAP	P01928	Hemoglobin subunit alpha OS=Cebus apella GN=HBA PE=1 SV=2	2476.57	15313.484	79	5	9	17.61	1153082	0	193037
162849	HBA_CALAR	P18972	Hemoglobin subunit alpha OS=Callithrix argentata GN=HBA PE=1 SV=1	2476.57	15196.312	79	5	8	17.73	1153082	129264	193037
162837	HBA_ATEGE	P67817	Hemoglobin subunit alpha OS=Ateles geoffroyi GN=HBA PE=1 SV=2	2476.57	15283.458	79	5	9	17.61	1153082	0	193037
162831	HBA_ANTPA	P14387	Hemoglobin subunit alpha OS=Antrozous pallidus GN=HBA PE=1 SV=2	2476.57	15354.524	79	5	9	17.61	1153082	0	193037
162729	HBA2_HYLLA	Q9TS34	Hemoglobin subunit alpha-2 OS=Hylobates lar GN=HBA2 PE=2 SV=2	2476.57	15384.563	79	5	9	17.61	1153082	0	193037

162696	HBA1_HYLLA	Q9TS35	Hemoglobin subunit alpha-1 OS=Hylobates lar GN=HBA1 PE=2 SV=2	2476.57	15325.539	79	5	9	17.61	1153082	0	193037
163393	HBD_LOXAF	Q45XJ0	Hemoglobin subunit delta OS=Loxodonta africana GN=HBD PE=2 SV=3	2027.076	16371.772	27	2	16	14.29	102923	0	15037
163388	HBD_ELEMA	Q45XI9	Hemoglobin subunit delta OS=Elephas maximus GN=HBD PE=2 SV=3	2027.076	16357.746	27	2	16	14.29	102923	0	15037
163366	HBB_TUPGL	P02052	Hemoglobin subunit beta OS=Tupaia glis GN=HBB PE=1 SV=1	2027.076	15971.238	27	2	10	14.38	102923	0	15037
163248	HBB_LOXAF	P02085	Hemoglobin subunit beta OS=Loxodonta africana GN=HBB PE=1 SV=1	2027.076	16240.574	27	2	15	14.38	102923	0	15037
163215	HBB_ELEMA	P02084	Hemoglobin subunit beta OS=Elephas maximus GN=HBB PE=1 SV=1	2027.076	16226.547	27	2	15	14.38	102923	0	15037
163213	HBB_ECHTE	P24292	Hemoglobin subunit beta OS=Echinops telfairi GN=HBB PE=1 SV=1	2027.076	16308.597	27	2	13	14.38	102923	0	15037
163544	HBG_OTOCR	P19760	Hemoglobin subunit gamma OS=Otolemur crassicaudatus GN=HBG PE=2 SV=3	1827.853	16301.778	34	4	13	27.89	210925	205143	52305
163402	HBD_TARSY	P13558	Hemoglobin subunit delta OS=Tarsius syrichta GN=HBD PE=2 SV=2	1765.712	16137.555	28	3	12	18.37	206190	0	51595
163401	HBD_SAISC	P02047	Hemoglobin subunit delta OS=Saimiri sciureus GN=HBD PE=1 SV=1	1765.712	15870.136	28	3	12	18.49	206190	0	51595
163400	HBD_SAGNI	P68015	Hemoglobin subunit delta OS=Saguinus nigricollis GN=HBD PE=1 SV=1	1765.712	15938.257	28	3	12	18.49	206190	0	51595
163399	HBD_SAGMY	P68014	Hemoglobin subunit delta OS=Saguinus mystax GN=HBD PE=1 SV=1	1765.712	15924.23	28	3	12	18.49	206190	0	51595
163394	HBD_OTOCR	Q03902	Hemoglobin subunit delta OS=Otolemur crassicaudatus GN=HBD PE=2 SV=2	1765.712	16202.559	28	3	13	18.37	206190	0	51595
163379	HBD_ATEGE	P02044	Hemoglobin subunit delta OS=Ateles geoffroyi GN=HBD PE=1 SV=3	1765.712	16049.465	28	3	13	18.37	206190	0	51595
163378	HBD_ATEFU	P33499	Hemoglobin subunit delta OS=Ateles fusciceps GN=HBD PE=1 SV=1	1765.712	15894.201	28	3	12	18.49	206190	0	51595
163377	HBD_AOTTR	P02046	Hemoglobin subunit delta OS=Aotus trivirgatus GN=HBD PE=1 SV=1	1765.712	15879.23	28	3	12	18.49	206190	0	51595
163368	HBB_TURTR	P18990	Hemoglobin subunit beta OS=Tursiops truncatus GN=HBB PE=1 SV=1	1765.712	16093.415	28	3	12	18.49	206190	0	51595
163357	HBB_TARSY	P13557	Hemoglobin subunit beta OS=Tarsius syrichta GN=HBB PE=2 SV=2	1765.712	16139.524	28	3	13	18.37	206190	0	51595
163356	HBB_TARBA	P02051	Hemoglobin subunit beta OS=Tarsius bancanus GN=HBB PE=1 SV=1	1765.712	15978.299	28	3	12	18.49	206190	0	51595
163355	HBB_TAPGE	P28781	Hemoglobin subunit beta OS=Taphozous georgianus GN=HBB PE=1 SV=1	1765.712	16018.298	28	3	12	18.49	206190	0	51595
163351	HBB_TALEU	P02061	Hemoglobin subunit beta OS=Talpa europaea GN=HBB PE=1 SV=1	1765.712	15903.228	28	3	12	18.49	206190	0	51595
163326	HBB_ROUAE	P02058	Hemoglobin subunit beta OS=Rousettus aegyptiacus GN=HBB PE=1 SV=1	1765.712	16071.401	28	3	12	18.49	206190	0	51595
163324	HBB_RHIFE	B2KHZ4	Hemoglobin subunit beta OS=Rhinolophus ferrumequinum GN=HBB PE=2 SV=1	1765.712	16139.542	28	3	13	18.37	206190	0	51595
163294	HBB_PANPO	P04244	Hemoglobin subunit beta OS=Panthera pardus orientalis GN=HBB PE=1 SV=2	1765.712	16233.489	28	3	11	18.37	206190	0	51595

163277	HBB_MYOVE	P11758	Hemoglobin subunit beta OS=Myotis velifer GN=HBB PE=1 SV=1	1765.712	15854.19	28	3	12	18.49	206190	0	51595
163264	HBB_MEGLY	P11752	Hemoglobin subunit beta OS=Megaderma lyra GN=HBB PE=1 SV=1	1765.712	16260.702	28	3	12	18.49	206190	0	51595
163255	HBB_MACGG	P24660	Hemoglobin subunit beta OS=Macroderma gigas GN=HBB PE=1 SV=1	1765.712	16042.363	28	3	12	18.49	206190	0	51595
163206	HBB_CYNSP	P11754	Hemoglobin subunit beta OS=Cynopterus sphinx GN=HBB PE=1 SV=1	1765.712	15985.318	28	3	12	18.49	206190	0	51595
163183	HBB_CEBCA	P67822	Hemoglobin subunit beta OS=Cebus capucinus GN=HBB PE=1 SV=1	1765.712	16105.332	28	3	12	18.49	206190	0	51595
163181	HBB_CEBAL	P02040	Hemoglobin subunit beta OS=Cebus albifrons GN=HBB PE=1 SV=1	1765.712	16075.306	28	3	12	18.49	206190	0	51595
163145	HBB_ANTPA	P14388	Hemoglobin subunit beta OS=Antrozous pallidus GN=HBB PE=1 SV=1	1765.712	16014.42	28	3	12	18.49	206190	0	51595
163136	HBB_ALCAA	P02073	Hemoglobin subunit beta OS=Alces alces alces GN=HBB PE=1 SV=1	1765.712	16280.643	28	3	12	18.62	206190	0	51595
163126	HBBF_SHEEP	P02083	Hemoglobin fetal subunit beta OS=Ovis aries PE=1 SV=1	1765.712	15989.307	28	3	12	18.62	206190	0	51595
163125	HBBF_CAPHI	P02082	Hemoglobin fetal subunit beta OS=Capra hircus PE=1 SV=1	1765.712	16004.318	28	3	12	18.62	206190	0	51595
163124	HBBF_BOVIN	P02081	Hemoglobin fetal subunit beta OS=Bos taurus PE=1 SV=1	1765.712	15975.264	28	3	12	18.62	206190	0	51595
163093	HBB2_TAPTE	P02065	Hemoglobin subunit beta-2 OS=Tapirus terrestris GN=HBB2 PE=1 SV=1	1765.712	16062.406	28	3	11	18.49	206190	0	51595
163088	HBB2_PANTS	P68194	Hemoglobin subunit beta-2 OS=Panthera tigris sumatrae GN=HBB2 PE=1 SV=1	1765.712	16072.265	28	3	10	18.49	206190	0	51595
163087	HBB2_PANPS	P68065	Hemoglobin subunit beta-2 OS=Panthera pardus saxicolor GN=HBB2 PE=1 SV=1	1765.712	16072.265	28	3	10	18.49	206190	0	51595
163086	HBB2_PANON	P68064	Hemoglobin subunit beta-2 OS=Panthera onca GN=HBB2 PE=1 SV=2	1765.712	16244.559	28	3	11	18.37	206190	0	51595
163085	HBB2_PANLE	P18988	Hemoglobin subunit beta-2 OS=Panthera leo GN=HBB2 PE=1 SV=2	1765.712	16044.251	28	3	10	18.49	206190	0	51595
163083	HBB2_MOUSE	P02089	Hemoglobin subunit beta-2 OS=Mus musculus GN=Hbb-b2 PE=1 SV=2	1765.712	15994.296	28	3	13	18.37	206190	0	51595
163075	HBB2_CHAMP	Q7M2Y5	Hemoglobin subunit beta-2 OS=Chalinolobus morio GN=HBB2 PE=1 SV=2	1765.712	15977.456	28	3	13	18.37	206190	0	51595
163061	HBB1_RAT	P02091	Hemoglobin subunit beta-1 OS=Rattus norvegicus GN=Hbb PE=1 SV=3	1765.712	16095.434	28	3	13	18.37	206190	0	51595
163059	HBB1_PANTS	P68048	Hemoglobin subunit beta-1 OS=Panthera tigris sumatrae GN=HBB1 PE=1 SV=2	1765.712	16233.489	28	3	11	18.37	206190	0	51595
163058	HBB1_PANPS	P68051	Hemoglobin subunit beta-1 OS=Panthera pardus saxicolor GN=HBB1 PE=1 SV=2	1765.712	16233.489	28	3	11	18.37	206190	0	51595
163057	HBB1_PANON	P68049	Hemoglobin subunit beta-1 OS=Panthera onca GN=HBB1 PE=1 SV=2	1765.712	16233.489	28	3	11	18.37	206190	0	51595
163056	HBB1_PANLE	P68050	Hemoglobin subunit beta-1 OS=Panthera leo GN=HBB1 PE=1 SV=1	1765.712	16102.291	28	3	10	18.49	206190	0	51595
163052	HBB1_MOUSE	P02088	Hemoglobin subunit beta-1 OS=Mus musculus GN=Hbb-b1 PE=1 SV=2	1765.712	15956.228	28	3	13	18.37	206190	0	51595

163043	HBB1_CHAMP	P68087	Hemoglobin subunit beta-1 OS=Chalinolobus morio GN=HBB1 PE=1 SV=2	1765.712	16022.453	28	3	13	18.37	206190	0	51595
163032	HBA_URSTH	P68236	Hemoglobin subunit alpha OS=Ursus thibetanus GN=HBA PE=1 SV=2	1762.991	15299.319	44	3	9	11.27	607728	0	103671
163031	HBA_URSMA	P68235	Hemoglobin subunit alpha OS=Ursus maritimus GN=HBA PE=1 SV=2	1762.991	15299.319	44	3	9	11.27	607728	0	103671
163028	HBA_TURTR	P18978	Hemoglobin subunit alpha OS=Tursiops truncatus GN=HBA PE=1 SV=1	1762.991	15402.571	44	3	9	11.35	607728	0	103671
162990	HBA_RABIT	P01948	Hemoglobin subunit alpha-1/2 OS=Oryctolagus cuniculus PE=1 SV=2	1762.991	15646.845	44	3	10	11.27	607728	0	103671
162987	HBA_PTEBR	P10885	Hemoglobin subunit alpha OS=Pteronura brasiliensis GN=HBA PE=1 SV=1	1762.991	15335.383	44	3	8	11.35	607728	0	103671
162983	HBA_PROLO	P18977	Hemoglobin subunit alpha OS=Procyon lotor GN=HBA PE=1 SV=1	1762.991	15272.346	44	3	8	11.35	607728	0	103671
162975	HBA_PHYCD	P09904	Hemoglobin subunit alpha OS=Physeter catodon GN=HBA PE=1 SV=1	1762.991	15291.451	44	3	9	11.35	607728	0	103671
162949	HBA_NASNA	P26915	Hemoglobin subunit alpha OS=Nasua nasua GN=HBA PE=1 SV=1	1762.991	15397.394	44	3	8	11.35	607728	0	103671
162947	HBA_MUSPU	P23601	Hemoglobin subunit alpha-1/2 OS=Mustela putorius PE=1 SV=1	1762.991	15347.353	44	3	8	11.35	607728	0	103671
162946	HBA_MUSPF	P20243	Hemoglobin subunit alpha-1/2 OS=Mustela putorius furo PE=1 SV=1	1762.991	15315.287	44	3	8	11.35	607728	0	103671
162945	HBA_MUSLU	P23600	Hemoglobin subunit alpha-1/2 OS=Mustela lutreola PE=1 SV=1	1762.991	15365.393	44	3	8	11.35	607728	0	103671
162939	HBA_MELME	P01953	Hemoglobin subunit alpha OS=Meles meles GN=HBA PE=1 SV=1	1762.991	15265.271	44	3	8	11.35	607728	0	103671
162904	HBA_HELMA	P68237	Hemoglobin subunit alpha OS=Helarctos malayanus GN=HBA PE=1 SV=2	1762.991	15299.319	44	3	9	11.27	607728	0	103671
162853	HBA_CANLA	P60530	Hemoglobin subunit alpha OS=Canis latrans GN=HBA PE=1 SV=2	1762.991	15494.568	44	3	9	11.27	607728	0	103671
162820	HBA_AILME	P18970	Hemoglobin subunit alpha OS=Ailuropoda melanoleuca GN=HBA PE=1 SV=2	1762.991	15277.357	44	3	9	11.27	607728	0	103671
162819	HBA_AILFU	P18969	Hemoglobin subunit alpha OS=Ailurus fulgens GN=HBA PE=1 SV=2	1762.991	15379.482	44	3	9	11.27	607728	0	103671
162718	HBA2_ARCGA	P41330	Hemoglobin subunit alpha-2 OS=Arctocephalus galapagoensis PE=1 SV=2	1762.991	15493.607	44	3	9	11.27	607728	0	103671
162684	HBA1_ARCGA	P41329	Hemoglobin subunit alpha-1 OS=Arctocephalus galapagoensis PE=1 SV=2	1762.991	15614.851	44	3	10	11.27	607728	0	103671
163702	HBT_PIG	P04246	Hemoglobin subunit theta OS=Sus scrofa PE=1 SV=1	1754.052	16038.447	26	2	10	13.01	205302	0	50633
163548	HBG_TARSY	P18436	Hemoglobin subunit gamma OS=Tarsius syrichta GN=HBG PE=2 SV=2	1754.052	16517.032	26	2	15	12.84	205302	0	50633
163547	HBG_TARBA	O77655	Hemoglobin subunit gamma OS=Tarsius bancanus GN=HBG PE=2 SV=3	1754.052	16398.913	26	2	15	12.93	205302	0	50633
163545	HBG_PAPCY	P68079	Hemoglobin subunit gamma OS=Papio cynocephalus GN=HBG PE=1 SV=2	1754.052	16159.427	26	2	13	12.93	205302	0	50633
163543	HBG_MACNE	P02097	Hemoglobin subunit gamma OS=Macaca nemestrina GN=HBG PE=1 SV=1	1754.052	16047.275	26	2	12	13.01	205302	0	50633

163542	HBG_MACMU	P68077	Hemoglobin subunit gamma OS=Macaca mulatta GN=HBG PE=1 SV=2	1754.052	16159.427	26	2	13	12.93	205302	0	50633
163541	HBG_MACFU	P68078	Hemoglobin subunit gamma OS=Macaca fuscata fuscata GN=HBG PE=1 SV=2	1754.052	16159.427	26	2	13	12.93	205302	0	50633
163540	HBG_LOXAF	Q45XH8	Hemoglobin subunit gamma OS=Loxodonta africana GN=HBG PE=2 SV=2	1754.052	16257.67	26	2	12	12.93	205302	0	50633
163539	HBG_LAGLA	P68070	Hemoglobin subunit gamma OS=Lagothrix lagotricha GN=HBG PE=2 SV=2	1754.052	15998.307	26	2	13	12.93	205302	0	50633
163537	HBG_ELEMA	Q45XH7	Hemoglobin subunit gamma OS=Elephas maximus GN=HBG PE=2 SV=3	1754.052	16287.696	26	2	12	12.93	205302	0	50633
163534	HBG_CALJA	Q9GJS7	Hemoglobin subunit gamma OS=Callithrix jacchus GN=HBG1 PE=2 SV=3	1754.052	16038.369	26	2	13	12.93	205302	0	50633
163533	HBG_BRAAR	P68071	Hemoglobin subunit gamma OS=Brachyteles arachnoides GN=HBG PE=2 SV=2	1754.052	15998.307	26	2	13	12.93	205302	0	50633
163532	HBG_ATEPA	P68069	Hemoglobin subunit gamma OS=Ateles paniscus GN=HBG PE=2 SV=2	1754.052	15998.307	26	2	13	12.93	205302	0	50633
163531	HBG_ATEGE	P68068	Hemoglobin subunit gamma OS=Ateles geoffroyi GN=HBG PE=2 SV=2	1754.052	15998.307	26	2	13	12.93	205302	0	50633
163530	HBG_AOTAZ	Q27940	Hemoglobin subunit gamma OS=Aotus azarae GN=HBG PE=2 SV=3	1754.052	15956.227	26	2	13	12.93	205302	0	50633
163529	HBG_ALOSE	P56285	Hemoglobin subunit gamma OS=Alouatta seniculus GN=HBG PE=2 SV=2	1754.052	15941.212	26	2	13	12.93	205302	0	50633
163528	HBG_ALOCA	P68031	Hemoglobin subunit gamma OS=Alouatta caraya GN=HBG PE=2 SV=2	1754.052	15957.255	26	2	13	12.93	205302	0	50633
163527	HBG_ALOBE	P68030	Hemoglobin subunit gamma OS=Alouatta belzebul GN=HBG PE=2 SV=2	1754.052	15957.255	26	2	13	12.93	205302	0	50633
163524	HBG2_HYLLA	P61948	Hemoglobin subunit gamma-2 OS=Hylobates lar GN=HBG2 PE=2 SV=2	1754.052	16129.444	26	2	13	12.93	205302	0	50633
163521	HBG2_CEBAP	P68257	Hemoglobin subunit gamma-2 OS=Cebus apella GN=HBG2 PE=2 SV=2	1754.052	15994.235	26	2	13	12.93	205302	0	50633
163520	HBG2_CEBAL	P68258	Hemoglobin subunit gamma-2 OS=Cebus albifrons GN=HBG2 PE=2 SV=2	1754.052	15994.235	26	2	13	12.93	205302	0	50633
163519	HBG1_PONPY	P18995	Hemoglobin subunit gamma-1 OS=Pongo pygmaeus GN=HBG1 PE=2 SV=2	1754.052	16189.453	26	2	13	12.93	205302	0	50633
163517	HBG1_PANPA	Q28779	Hemoglobin subunit gamma-1 OS=Pan paniscus GN=HBG1 PE=2 SV=3	1754.052	16226.518	26	2	13	12.93	205302	0	50633
163516	HBG1_HYLLA	P61947	Hemoglobin subunit gamma-1 OS=Hylobates lar GN=HBG1 PE=2 SV=2	1754.052	16101.391	26	2	13	12.93	205302	0	50633
163514	HBG1_GORGO	P62741	Hemoglobin subunit gamma-1 OS=Gorilla gorilla gorilla GN=HBG1 PE=2 SV=2	1754.052	16168.481	26	2	13	12.93	205302	0	50633
163513	HBG1_CEBAP	P68256	Hemoglobin subunit gamma-1 OS=Cebus apella GN=HBG1 PE=2 SV=2	1754.052	15980.208	26	2	13	12.93	205302	0	50633
163512	HBG1_CEBAL	Q28221	Hemoglobin subunit gamma-1 OS=Cebus albifrons GN=HBG1 PE=2 SV=3	1754.052	15980.208	26	2	13	12.93	205302	0	50633
163511	HBG1_CALMO	Q9GLX4	Hemoglobin subunit gamma-1 OS=Callicebus moloch GN=HBG1 PE=2 SV=3	1754.052	16012.291	26	2	13	12.93	205302	0	50633
163509	HBE_TARSY	P18435	Hemoglobin subunit epsilon OS=Tarsius syrichta GN=HBE1 PE=2 SV=2	1754.052	16235.725	26	2	13	12.93	205302	0	50633

163508	HBE_SMICR	Q28931	Hemoglobin subunit epsilon OS=Sminthopsis crassicaudata GN=HBE1 PE=2 SV=3	1754.052	16250.651	26	2	13	12.93	205302	0	50633
163507	HBE_SAISC	P68028	Hemoglobin subunit epsilon OS=Saimiri sciureus GN=HBE1 PE=2 SV=2	1754.052	16349.915	26	2	13	12.93	205302	0	50633
163506	HBE_SAIBB	P68029	Hemoglobin subunit epsilon OS=Saimiri boliviensis boliviensis GN=HBE1 PE=2 SV=2	1754.052	16349.915	26	2	13	12.93	205302	0	50633
163505	HBE_SAGMI	P51443	Hemoglobin subunit epsilon OS=Saguinus midas GN=HBE1 PE=2 SV=2	1754.052	16361.948	26	2	13	12.93	205302	0	50633
163501	HBE_PITIR	P51442	Hemoglobin subunit epsilon OS=Pithecia irrorata GN=HBE1 PE=2 SV=2	1754.052	16335.932	26	2	13	12.93	205302	0	50633
163500	HBE_PIG	P02101	Hemoglobin subunit epsilon OS=Sus scrofa GN=HBE1 PE=1 SV=3	1754.052	16152.662	26	2	11	12.93	205302	0	50633
163493	HBE_MACEU	P81042	Hemoglobin subunit epsilon OS=Macropus eugenii GN=HBE1 PE=1 SV=3	1754.052	16133.566	26	2	13	12.93	205302	0	50633
163492	HBE_LEORO	P51441	Hemoglobin subunit epsilon OS=Leontopithecus rosalia GN=HBE1 PE=2 SV=2	1754.052	16335.889	26	2	13	12.93	205302	0	50633
163491	HBE_LAGLA	P51440	Hemoglobin subunit epsilon OS=Lagothrix lagotricha GN=HBE1 PE=2 SV=2	1754.052	16391.953	26	2	13	12.93	205302	0	50633
163486	HBE_DIDVI	P11025	Hemoglobin subunit epsilon-M OS=Didelphis virginiana GN=HBE1 PE=2 SV=2	1754.052	16319.689	26	2	13	12.93	205302	0	50633
163485	HBE_DAUMA	Q28356	Hemoglobin subunit epsilon OS=Daubentonia madagascariensis GN=HBE1 PE=2 SV=3	1754.052	16225.757	26	2	13	12.93	205302	0	50633
163484	HBE_CHISA	P68025	Hemoglobin subunit epsilon OS=Chiropotes satanas GN=HBE1 PE=2 SV=2	1754.052	16319.889	26	2	13	12.93	205302	0	50633
163481	HBE_CEBOL	Q29415	Hemoglobin subunit epsilon OS=Cebus olivaceus GN=HBE1 PE=2 SV=3	1754.052	16377.969	26	2	13	12.93	205302	0	50633
163480	HBE_CEBKA	Q7JFR7	Hemoglobin subunit epsilon OS=Cebus kaapori GN=HBE1 PE=2 SV=3	1754.052	16377.969	26	2	13	12.93	205302	0	50633
163479	HBE_CEBAL	Q28220	Hemoglobin subunit epsilon OS=Cebus albifrons GN=HBE1 PE=2 SV=3	1754.052	16363.942	26	2	13	12.93	205302	0	50633
163478	HBE_CALTO	P68023	Hemoglobin subunit epsilon OS=Callicebus torquatus GN=HBE1 PE=2 SV=2	1754.052	16319.889	26	2	13	12.93	205302	0	50633
163477	HBE_CALPY	P68027	Hemoglobin subunit epsilon OS=Callithrix pygmaea GN=HBE1 PE=2 SV=2	1754.052	16301.85	26	2	13	12.93	205302	0	50633
163476	HBE_CALMO	P68022	Hemoglobin subunit epsilon OS=Callicebus moloch GN=HBE1 PE=2 SV=2	1754.052	16319.889	26	2	13	12.93	205302	0	50633
163475	HBE_CALJA	P68026	Hemoglobin subunit epsilon OS=Callithrix jacchus GN=HBE1 PE=2 SV=2	1754.052	16301.85	26	2	13	12.93	205302	0	50633
163474	HBE_CALGO	P68024	Hemoglobin subunit epsilon OS=Callimico goeldii GN=HBE1 PE=2 SV=2	1754.052	16319.889	26	2	13	12.93	205302	0	50633
163473	HBE_CALGE	O13071	Hemoglobin subunit epsilon (Fragment) OS=Callithrix geoffroyi GN=HBE1 PE=2 SV=3	1754.052	15580.03	26	2	13	13.38	205302	0	50633
163471	HBE_CACCA	P68021	Hemoglobin subunit epsilon OS=Cacajao calvus GN=HBE1 PE=2 SV=2	1754.052	16319.889	26	2	13	12.93	205302	0	50633
163470	HBE_BRATR	Q45XH3	Hemoglobin subunit epsilon OS=Bradypus tridactylus GN=HBE1 PE=2 SV=3	1754.052	16213.597	26	2	13	12.93	205302	0	50633
163469	HBE_BRAAR	P68020	Hemoglobin subunit epsilon OS=Brachyteles arachnoides GN=HBE1 PE=2 SV=2	1754.052	16319.889	26	2	13	12.93	205302	0	50633

163468	HBE_ATEGE	P68019	Hemoglobin subunit epsilon OS=Ateles geoffroyi GN=HBE1 PE=2 SV=2	1754.052	16319.889	26	2	13	12.93	205302	0	50633
163467	HBE_ATEBE	P51438	Hemoglobin subunit epsilon OS=Ateles belzebuth GN=HBE1 PE=2 SV=2	1754.052	16285.872	26	2	13	12.93	205302	0	50633
163466	HBE_AOTAZ	P68016	Hemoglobin subunit epsilon OS=Aotus azarae GN=HBE1 PE=2 SV=2	1754.052	16319.889	26	2	13	12.93	205302	0	50633
163465	HBE_ALOSE	P68018	Hemoglobin subunit epsilon OS=Alouatta seniculus GN=HBE1 PE=2 SV=2	1754.052	16319.889	26	2	13	12.93	205302	0	50633
163464	HBE_ALOBE	P68017	Hemoglobin subunit epsilon OS=Alouatta belzebul GN=HBE1 PE=2 SV=2	1754.052	16319.889	26	2	13	12.93	205302	0	50633
163405	HBE1_CAPHI	P02102	Hemoglobin subunit epsilon-1 OS=Capra hircus GN=HBE1 PE=2 SV=2	1754.052	16128.574	26	2	13	12.93	205302	0	50633
163334	HBB_SCAOR	Q2KPA3	Hemoglobin subunit beta OS=Scapanus orarius GN=HBB PE=2 SV=3	1754.052	16072.543	26	2	13	12.93	205302	0	50633
163333	HBB_SCAAQ	Q2KPA4	Hemoglobin subunit beta OS=Scalopus aquaticus GN=HBB PE=2 SV=3	1754.052	16310.856	26	2	13	12.93	205302	0	50633
163322	HBB_RANTA	P21380	Hemoglobin subunit beta OS=Rangifer tarandus GN=HBB PE=1 SV=1	1754.052	16224.579	26	2	12	13.1	205302	0	50633
163313	HBB_PROHA	P02086	Hemoglobin subunit beta OS=Procavia capensis habessinica GN=HBB PE=1 SV=1	1754.052	16263.507	26	2	12	13.01	205302	0	50633
163283	HBB_ODOVI	P02074	Hemoglobin subunit beta-3 OS=Odocoileus virginianus virginianus GN=HBB PE=1 SV=1	1754.052	15882.227	26	2	11	13.1	205302	0	50633
163271	HBB_MICPE	B3EWE4	Hemoglobin subunit beta OS=Microtus pennsylvanicus PE=1 SV=1	1754.052	15735.05	26	2	12	13.01	205302	0	50633
163251	HBB_MACCA	P09840	Hemoglobin subunit beta OS=Macrotus californicus GN=HBB PE=1 SV=1	1754.052	16064.333	26	2	12	13.01	205302	0	50633
163218	HBB_ERIEU	P02059	Hemoglobin subunit beta OS=Erinaceus europaeus GN=HBB PE=1 SV=1	1754.052	15921.288	26	2	12	13.01	205302	0	50633
163180	HBB_CAVPO	P02095	Hemoglobin subunit beta OS=Cavia porcellus GN=HBB PE=1 SV=1	1754.052	16037.37	26	2	10	13.01	205302	0	50633
163161	HBB_BLABR	B3EWE2	Hemoglobin subunit beta OS=Blarina brevicauda PE=1 SV=1	1754.052	15853.307	26	2	12	13.01	205302	0	50633
163121	HBBC_SHEEP	P68056	Hemoglobin subunit beta-C OS=Ovis aries GN=HBBC PE=1 SV=2	1754.052	15808.12	26	2	11	13.38	205302	0	50633
163120	HBBC_OVIMU	P68057	Hemoglobin subunit beta-C OS=Ovis orientalis musimon GN=HBBC PE=1 SV=1	1754.052	15676.922	26	2	11	13.48	205302	0	50633
163114	HBBC_CAPHI	P02078	Hemoglobin subunit beta-C OS=Capra hircus GN=HBBC PE=2 SV=2	1754.052	15809.105	26	2	11	13.38	205302	0	50633
163112	HBBC_AMMLE	P68058	Hemoglobin subunit beta-C OS=Ammotragus lervia PE=1 SV=1	1754.052	15562.818	26	2	11	13.48	205302	0	50633
163090	HBB2_RAT	P11517	Hemoglobin subunit beta-2 OS=Rattus norvegicus PE=1 SV=2	1754.052	16098.481	26	2	13	12.93	205302	0	50633
163024	HBA_TRIIN	P07414	Hemoglobin subunit alpha OS=Trichechus inunguis GN=HBA PE=1 SV=1	1742.794	15704.772	64	4	11	12.77	835766	0	143744
163022	HBA_TRAST	P04237	Hemoglobin subunit alpha OS=Tragelaphus strepsiceros GN=HBA PE=1 SV=1	1742.794	14949.045	64	4	8	12.86	835766	0	143744
163020	HBA_THEGE	P01932	Hemoglobin subunit alpha OS=Theropithecus gelada GN=HBA PE=1 SV=1	1742.794	15550.846	64	4	12	12.77	835766	0	143744

163013	HBA_TAMHU	B3EWD7	Hemoglobin subunit alpha OS=Tamiasciurus hudsonicus PE=1 SV=1	1742.794	15044.157	64	4	9	12.77	835766	0	143744
163002	HBA_SHEEP	P68240	Hemoglobin subunit alpha-1/2 OS=Ovis aries PE=1 SV=2	1742.794	15222.388	64	4	10	12.68	835766	0	143744
163000	HBA_SCICA	B3EWD1	Hemoglobin subunit alpha OS=Sciurus carolinensis PE=1 SV=1	1742.794	15131.278	64	4	8	12.77	835766	0	143744
162995	HBA_ROUAE	P01956	Hemoglobin subunit alpha OS=Rousettus aegyptiacus GN=HBA PE=1 SV=1	1742.794	15191.261	64	4	8	12.77	835766	0	143744
162994	HBA_RHIUN	P09906	Hemoglobin subunit alpha OS=Rhinoceros unicornis GN=HBA PE=1 SV=1	1742.794	15377.477	64	4	8	12.77	835766	0	143744
162991	HBA_RANTA	P21379	Hemoglobin subunit alpha OS=Rangifer tarandus GN=HBA PE=1 SV=1	1742.794	15101.236	64	4	9	12.77	835766	0	143744
162989	HBA_PTEPO	P14390	Hemoglobin subunit alpha OS=Pteropus poliocephalus GN=HBA PE=1 SV=1	1742.794	15280.379	64	4	8	12.77	835766	0	143744
162988	HBA_PTEGI	D0VX09	Hemoglobin subunit alpha OS=Pteropus giganteus GN=HBA PE=1 SV=1	1742.794	15208.288	64	4	8	12.77	835766	0	143744
162986	HBA_PTEAL	P14389	Hemoglobin subunit alpha OS=Pteropus alecto GN=HBA PE=1 SV=1	1742.794	15231.325	64	4	8	12.77	835766	0	143744
162982	HBA_PROHA	P01957	Hemoglobin subunit alpha OS=Procavia capensis habessinica GN=HBA PE=1 SV=1	1742.794	15633.742	64	4	10	12.68	835766	0	143744
162967	HBA_PAPCY	P63112	Hemoglobin subunit alpha OS=Papio cynocephalus GN=HBA PE=1 SV=2	1742.794	15624.949	64	4	11	12.68	835766	0	143744
162966	HBA_PAPAN	P63111	Hemoglobin subunit alpha OS=Papio anubis GN=HBA PE=2 SV=2	1742.794	15624.949	64	4	11	12.68	835766	0	143744
162958	HBA_PANHO	Q0ZA50	Hemoglobin subunit alpha OS=Pantholops hodgsonii GN=HBA PE=2 SV=3	1742.794	15179.363	64	4	10	12.68	835766	0	143744
162936	HBA_MEGLY	P11751	Hemoglobin subunit alpha OS=Megaderma lyra GN=HBA PE=1 SV=1	1742.794	14817.992	64	4	8	12.77	835766	0	143744
162923	HBA_MACCA	P09839	Hemoglobin subunit alpha OS=Macrotus californicus GN=HBA PE=1 SV=1	1742.794	15190.302	64	4	8	12.77	835766	0	143744
162906	HBA_HIPAM	P19015	Hemoglobin subunit alpha OS=Hippopotamus amphibius GN=HBA PE=1 SV=1	1742.794	15308.377	64	4	9	12.77	835766	0	143744
162877	HBA_CTEGU	P20854	Hemoglobin subunit alpha OS=Ctenodactylus gundi GN=HBA PE=1 SV=1	1742.794	15268.345	64	4	8	12.77	835766	0	143744
162861	HBA_CERSI	P01963	Hemoglobin subunit alpha OS=Ceratotherium simum GN=HBA PE=1 SV=2	1742.794	15492.676	64	4	9	12.68	835766	0	143744
162860	HBA_CERAT	P01933	Hemoglobin subunit alpha OS=Cercocebus atys GN=HBA PE=1 SV=1	1742.794	15444.725	64	4	10	12.77	835766	0	143744
162843	HBA_BOSGF	P01969	Hemoglobin subunit alpha OS=Bos gaurus frontalis GN=HBA PE=1 SV=2	1742.794	15197.391	64	4	10	12.68	835766	0	143744
162824	HBA_AMMLE	P68239	Hemoglobin subunit alpha-1/2 OS=Ammotragus lervia PE=1 SV=2	1742.794	15222.388	64	4	10	12.68	835766	0	143744
162821	HBA_ALCAA	P01971	Hemoglobin subunit alpha OS=Alces alces alces GN=HBA PE=1 SV=2	1742.794	15246.461	64	4	10	12.68	835766	0	143744
162752	HBA4_BUBBU	Q9XSK1	Hemoglobin subunit alpha-4 OS=Bubalus bubalis PE=2 SV=3	1742.794	15171.354	64	4	10	12.68	835766	0	143744
162745	HBA3_BUBBU	Q9TSN9	Hemoglobin subunit alpha-3 OS=Bubalus bubalis PE=2 SV=3	1742.794	15189.328	64	4	11	12.68	835766	0	143744

162732	HBA2_OTOCR	P01939	Hemoglobin subunit alpha-B OS=Otolemur crassicaudatus GN=HBAB PE=1 SV=3	1742.794	15635.903	64	4	9	12.68	835766	0	143744
162723	HBA2_CAPHI	P0CH26	Hemoglobin subunit alpha-2 OS=Capra hircus GN=HBA2 PE=1 SV=1	1742.794	15249.371	64	4	10	12.68	835766	0	143744
162722	HBA2_BUBBU	Q9TSN8	Hemoglobin subunit alpha-2 OS=Bubalus bubalis PE=1 SV=3	1742.794	15185.337	64	4	11	12.68	835766	0	143744
162709	HBA1_TADBR	P11755	Hemoglobin subunit alpha-1 OS=Tadarida brasiliensis PE=1 SV=1	1742.794	15368.434	64	4	8	12.77	835766	0	143744
162695	HBA1_HAPGR	Q7M3B8	Hemoglobin subunit alpha-1 OS=Hapalemur griseus GN=HBA1 PE=1 SV=1	1742.794	15070.135	64	4	9	12.86	835766	0	143744
162689	HBA1_CAPHI	P0CH25	Hemoglobin subunit alpha-1 OS=Capra hircus GN=HBA1 PE=1 SV=1	1742.794	15222.388	64	4	10	12.68	835766	0	143744
162688	HBA1_BUBBU	Q9TSN7	Hemoglobin subunit alpha-1 OS=Bubalus bubalis PE=2 SV=3	1742.794	15128.285	64	4	11	12.68	835766	0	143744
163375	HBB_VULVU	P21201	Hemoglobin subunit beta OS=Vulpes vulpes GN=HBB PE=1 SV=1	1683.261	16096.408	22	2	12	13.7	91214	0	12717
163257	HBB_MACMU	P02026	Hemoglobin subunit beta OS=Macaca mulatta GN=HBB PE=1 SV=1	1683.261	16113.355	22	2	12	13.7	91214	0	12717
163192	HBB_CHRBR	P60526	Hemoglobin subunit beta OS=Chrysocyon brachyurus GN=HBB PE=1 SV=1	1683.261	16112.408	22	2	12	13.7	91214	0	12717
163177	HBB_CANLA	P60525	Hemoglobin subunit beta OS=Canis latrans GN=HBB PE=1 SV=1	1683.261	16112.408	22	2	12	13.7	91214	0	12717
163176	HBB_CANFA	P60524	Hemoglobin subunit beta OS=Canis familiaris GN=HBB PE=1 SV=1	1683.261	16112.408	22	2	12	13.7	91214	0	12717
162748	HBA3_PANTR	P01935	Hemoglobin subunit alpha-3 OS=Pan troglodytes PE=1 SV=1	1447.356	14207.519	50	3	5	11.35	862670	0	138659
162746	HBA3_GORGO	P01934	Hemoglobin subunit alpha-3 OS=Gorilla gorilla gorilla PE=1 SV=1	1447.356	14336.635	50	3	5	11.35	862670	0	138659
163350	HBB_TADBR	P11756	Hemoglobin subunit beta OS=Tadarida brasiliensis GN=HBB PE=1 SV=1	1410.237	15790.097	21	2	12	12.33	193593	0	48313
163290	HBB_PAGLA	P19646	Hemoglobin subunit beta OS=Paguma larvata GN=HBB PE=1 SV=1	1410.237	16127.385	21	2	12	12.33	193593	0	48313
163274	HBB_MUSLU	P23602	Hemoglobin subunit beta OS=Mustela lutreola GN=HBB PE=1 SV=1	1410.237	16051.367	21	2	12	12.33	193593	0	48313
163160	HBB_BISBO	P09422	Hemoglobin subunit beta OS=Bison bonasus GN=HBB PE=1 SV=1	1410.237	16034.395	21	2	12	12.41	193593	0	48313
163538	HBG_EULFU	P08225	Hemoglobin subunit gamma OS=Eulemur fulvus fulvus GN=HBG PE=2 SV=2	1398.577	16291.835	19	1	13	6.8	192705	0	47351
163525	HBG2_PANTR	P61921	Hemoglobin subunit gamma-2 OS=Pan troglodytes GN=HBG2 PE=1 SV=2	1398.577	16184.477	19	1	13	6.8	192705	0	47351
163523	HBG2_HUMAN	P69892	Hemoglobin subunit gamma-2 OS=Homo sapiens GN=HBG2 PE=1 SV=2	1398.577	16184.477	19	1	13	6.8	192705	0	47351
163522	HBG2_GORGO	P62742	Hemoglobin subunit gamma-2 OS=Gorilla gorilla gorilla GN=HBG2 PE=2 SV=2	1398.577	16184.477	19	1	13	6.8	192705	0	47351
163518	HBG1_PANTR	P61920	Hemoglobin subunit gamma-1 OS=Pan troglodytes GN=HBG1 PE=1 SV=2	1398.577	16198.504	19	1	13	6.8	192705	0	47351

163515	HBG1_HUMAN	P69891	Hemoglobin subunit gamma-1 OS=Homo sapiens GN=HBG1 PE=1 SV=2	1398.577	16198.504	19	1	13	6.8	192705	0	47351
163369	HBB_UROTO	P07410	Hemoglobin subunit beta-S/F OS=Urocitellus townsendii PE=1 SV=1	1398.577	15838.103	19	1	12	6.85	192705	0	47351
163033	HBA_VICPA	P67816	Hemoglobin subunit alpha OS=Vicugna pacos GN=HBA PE=1 SV=1	1029.215	15184.321	29	2	9	6.38	290412	0	54378
163027	HBA_TURME	P14522	Hemoglobin subunit alpha-A OS=Turdus merula GN=HBAA PE=1 SV=1	1029.215	15163.322	29	2	9	6.38	290412	0	54378
163025	HBA_TRIOC	P19832	Hemoglobin subunit alpha-A OS=Trigonoceps occipitalis GN=HBAA PE=1 SV=1	1029.215	15583.929	29	2	9	6.38	290412	0	54378
163017	HBA_TAPTE	P01962	Hemoglobin subunit alpha-1/2 OS=Tapirus terrestris PE=1 SV=1	1029.215	15348.436	29	2	9	6.38	290412	0	54378
163012	HBA_TALEU	P01951	Hemoglobin subunit alpha OS=Talpa europaea GN=HBA PE=1 SV=1	1029.215	15304.289	29	2	8	6.38	290412	0	54378
163011	HBA_SUNMU	P01950	Hemoglobin subunit alpha OS=Suncus murinus GN=HBA PE=1 SV=1	1029.215	15040.098	29	2	9	6.38	290412	0	54378
163003	HBA_SPAEH	P01943	Hemoglobin subunit alpha OS=Spalax ehrenbergi GN=HBA PE=1 SV=1	1029.215	15318.288	29	2	8	6.38	290412	0	54378
162992	HBA_RAT	P01946	Hemoglobin subunit alpha-1/2 OS=Rattus norvegicus GN=Hba1 PE=1 SV=3	1029.215	15502.589	29	2	10	6.34	290412	0	54378
162970	HBA_PERCR	B3EWD3	Hemoglobin subunit alpha OS=Peromyscus crinitus PE=1 SV=1	1029.215	15044.052	29	2	8	6.38	290412	0	54378
162969	HBA_PERCA	B3EWD5	Hemoglobin subunit alpha OS=Peromyscus californicus PE=1 SV=1	1029.215	14984.974	29	2	8	6.38	290412	0	54378
162954	HBA_ORNAN	P01979	Hemoglobin subunit alpha OS=Ornithorhynchus anatinus GN=HBA PE=1 SV=1	1029.215	15450.566	29	2	12	6.38	290412	0	54378
162953	HBA_ONDZI	P01944	Hemoglobin subunit alpha OS=Ondatra zibethicus GN=HBA PE=1 SV=1	1029.215	15214.345	29	2	8	6.38	290412	0	54378
162943	HBA_MOUSE	P01942	Hemoglobin subunit alpha OS=Mus musculus GN=Hba PE=1 SV=2	1029.215	15143.206	29	2	9	6.34	290412	0	54378
162941	HBA_MICPE	B3EWE3	Hemoglobin subunit alpha OS=Microtus pennsylvanicus PE=1 SV=1	1029.215	15131.211	29	2	8	6.38	290412	0	54378
162940	HBA_MESAU	P01945	Hemoglobin subunit alpha OS=Mesocricetus auratus GN=HBA PE=1 SV=1	1029.215	15306.36	29	2	9	6.38	290412	0	54378
162937	HBA_MELCA	P15448	Hemoglobin subunit alpha OS=Mellivora capensis GN=HBA PE=1 SV=1	1029.215	15337.355	29	2	8	6.38	290412	0	54378
162912	HBA_LAMVI	P07425	Hemoglobin subunit alpha OS=Lama vicugna GN=HBA PE=1 SV=1	1029.215	15200.321	29	2	9	6.38	290412	0	54378
162911	HBA_LAMGU	P67815	Hemoglobin subunit alpha OS=Lama guanicoe GN=HBA PE=1 SV=1	1029.215	15184.321	29	2	9	6.38	290412	0	54378
162910	HBA_LAMGL	P01973	Hemoglobin subunit alpha OS=Lama glama GN=HBA PE=1 SV=1	1029.215	15162.269	29	2	9	6.38	290412	0	54378
162903	HBA_GYPRU	P08256	Hemoglobin subunit alpha-A/A' OS=Gyps rueppellii GN=HBAA PE=1 SV=1	1029.215	15598.9	29	2	9	6.38	290412	0	54378
162886	HBA_DIDVI	P01976	Hemoglobin subunit alpha OS=Didelphis virginiana GN=HBA PE=1 SV=1	1029.215	15315.42	20	1	10	4.96	282821	0	51392

162884	HBA_DASNO	P01964	Hemoglobin subunit alpha OS=Dasypus novemcinctus GN=HBA PE=1 SV=1	1029.215	15295.566	29	2	8	6.38	290412	0	54378
162873	HBA_CRIGA	P19014	Hemoglobin subunit alpha OS=Cricetomys gambianus GN=HBA PE=1 SV=2	1029.215	15237.363	29	2	9	6.34	290412	0	54378
162842	HBA_BLABR	B3EWE1	Hemoglobin subunit alpha OS=Blarina brevicauda PE=1 SV=1	1029.215	15053.114	29	2	9	6.38	290412	0	54378
162818	HBA_AEGMO	P07417	Hemoglobin subunit alpha-A OS=Aegypius monachus GN=HBAA PE=1 SV=2	1029.215	15729.154	29	2	10	6.34	290412	0	54378
162793	HBAD_RHEAM	P04241	Hemoglobin subunit alpha-D OS=Rhea americana GN=HBAD PE=1 SV=1	1029.215	15846.095	29	2	13	6.38	290412	0	54378
163526	HBG2_PONPY	P18996	Hemoglobin subunit gamma-2 OS=Pongo pygmaeus GN=HBG2 PE=2 SV=3	355.4748	16203.48	7	1	13	6.12	12597	0	3282
89503	DCD_HUMAN	P81605	Dermcidin OS=Homo sapiens GN=DCD PE=1 SV=2	214.7067	11399.902	14	3	11	26.36	14915	21165	3589
50457	CASA1_BUBBU	O62823	Alpha-S1-casein OS=Bubalus bubalis GN=CSN1S1 PE=2 SV=2	164.4248	24384.828	16	3	16	13.55	7749	0	2472
50456	CASA1_BOVIN	P02662	Alpha-S1-casein OS=Bos taurus GN=CSN1S1 PE=1 SV=2	164.4248	24586.997	16	3	17	13.55	7749	7749	2472
476496	TTHY_HUMAN	P02766	Transthyretin OS=Homo sapiens GN=TTR PE=1 SV=1	74.7515	16003.066	11	3	9	24.49	3881	4516.5	2216
476503	TTHY_PANTR	Q5U7I5	Transthyretin OS=Pan troglodytes GN=TTR PE=2 SV=1	56.9412	15988.135	7	2	9	15.65	1812	0	1661
21639	ARHG2_PIG	B2DCZ9	Rho guanine nucleotide exchange factor 2 OS=Sus scrofa GN=ARHGEF2 PE=2 SV=1	49.3546	109295.41	17	5	97	4.99	18567	29496	3442
174137	HOIL1_DANRE	A9JTG5	RanBP-type and C3HC4-type zinc finger-containing protein 1 OS=Danio rerio GN=rbck1 PE=2 SV=1	46.5159	81465.016	30	9	60	4.76	12752	3777	8409
12516	ALBU_BOVIN	P02769	Serum albumin OS=Bos taurus GN=ALB PE=1 SV=4	42.4383	71323.87	28	9	59	12.19	14133	7806	5876
479296	U483_DROPS	Q29BR3	UPF0483 protein GA18864 OS=Drosophila pseudoobscura pseudoobscura GN=GA18864 PE=3 SV=1	36.9831	31541.859	8	2	16	14.53	2690	4035	1604
433236	SSG1_IPOBA	Q42857	Granule-bound starch synthase 1_ chloroplastic/amyloplastic OS=Ipomoea batatas GN=WAXY PE=2 SV=2	34.6475	67675.337	12	3	49	5.43	12013	11970	3037
316266	PTN4_MOUSE	Q9WU22	Tyrosine-protein phosphatase non-receptor type 4 OS=Mus musculus GN=Ptpn4 PE=1 SV=2	34.3106	106992.27	16	6	73	4.64	11555	10561.5	5510
