# Supplementary material for: Free serum haemoglobin is associated with brain atrophy in secondary progressive multiple sclerosis
Source: Wellcome Open Res. 2016 Dec 23;1:10. Originally published 2016 Nov 15. [Version 2] doi: 10.12688/wellcomeopenres.9967.2 (PMC5159626; doi:10.12688/wellcomeopenres.9967.2)
Supplement: Peer review history

# Peer review: Referee response for version 2

**DOI:** 10.21956/wellcomeopenres.11315.r18853

**Published:** 2016-12-30

**Authors:** George Harauz, Vladimir V. Bamm

**Affiliations:** 1 Department of Molecular and Cellular Biology, University of Guelph, Guelph, ON, Canada

We are happy to approve this article in in its revised form. The authors responded to all points of concern and their contribution is an important one.

We have read this submission. We believe that we have an appropriate level of expertise to confirm that it is of an acceptable scientific standard.

# Peer review: Referee response for version 2

**DOI:** 10.21956/wellcomeopenres.11315.r18826

**Published:** 2016-12-28

**Authors:** Franz Fazekas, Michael Khalil

**Affiliations:** 1 Department of Neurology, Medical University of Graz, Graz, Austria; 2 Department of Neurology, Department of Neurology, Medical University of Graz, Graz, Austria, Graz, Austria

The authors have adequately addressed all our comments, questions and suggestions.

We have read this submission. We believe that we have an appropriate level of expertise to confirm that it is of an acceptable scientific standard.

# Peer review: Referee response for version 1

**DOI:** 10.21956/wellcomeopenres.10742.r17822

**Published:** 2016-12-12

**Authors:** Franz Fazekas, Michael Khalil

**Affiliations:** 1 Department of Neurology, Medical University of Graz, Graz, Austria; 2 Department of Neurology, Department of Neurology, Medical University of Graz, Graz, Austria, Graz, Austria

This is an interesting study linking an increase of serum hemoglobin to brain atrophy in patients with secondary progressive multiple sclerosis. In this study 140 patients have been included, who were initially recruited for the phase 2, placebo-controlled MS-STAT trial, investigating the effect of simvastatin on brain volume loss after 2 years of treatment. A total of 475 serum samples collected at baseline, 6 months, 12 and 24 months have been analyzed using an unbiased search for protein profiles by SELDI-TOF mass spectrometry. Brain volume changes were measured by MRI Brain Boundary Shift Integral (BBSI) at baseline, 12 months and 25 months.

 An increased abundance of serum proteins at 15.1 kDa and 15.9 kDa were significantly correlated to the rate of brain atrophy after 2 years. These proteins have then been identified as alpha-hemoglobin and beta hemoglobin using tandem mass spectrometry. In addition, higher serum hemoglobin concentration in SPMS patients compared to controls has been confirmed using ELISA. The authors further studied serum lactate dehydrogenase activity, which has also been shown to be significantly increased in SPMS compared to controls.

 It was concluded that a low-grade chronic intravascular hemolysis, which is a potential source of iron whose deposition along blood vessels in multiple sclerosis plaques contributes to neurodegeneration and brain atrophy in progressive MS.

 The results of this study are intriguing and underline other reports on the possible implication of hemoglobin in the pathogenesis of multiple sclerosis, reviewed in Altinoz *et al.* (2016). Lewin *et al.* hypothesize that increased serum levels of free hemoglobin may trigger iron deposition along blood vessels in MS, which could then propagate neurodegeneration and brain atrophy. While this speculation brings in a new idea, it appears biologically difficult how these processes (e.g. iron deposition along blood vessels in MS lesions and cortical atrophy) should be so tightly linked in time if at all. Otherwise increased iron deposition has been shown to occur in MS using several MRI techniques although due to technical reasons robust quantification of brain iron deposition is currently limited to deep gray matter areas (Ropele *et al.* 2011). Longitudinal studies are still scarce, but a recent report indicates that increased brain iron deposition is more pronounced in early phases of the disease (Khalil *et al.* 2015). Less information exists on progressive forms of MS but one would speculate that the damaging effects from iron deposition are a long-standing rather than an immediate effect as suggested by Lewin *et al.*. Observed correlation thus are likely not able to clarify if and to which extent increased brain iron deposition really amplifies neurodegeneration in MS or merely reflects an epiphenomenon of the disease. There is also some evidence for alterations of iron related proteins in CSF and serum of MS patients, including transferrin (Khalil *et al.* 2014)  and lipocalin 2 (Khalil *et al.* 2016), and which may contribute to iron accumulation.

 Reiterating some of the concerns and comments of the other reviewers we thus see the need to put reported findings in a more cautious context. Also some methodological issues deserve clarification.

**Major comments**

- The authors should rather use the term “correlated” instead of “associated” and discuss that presented observations do not prove causality as outlined above.
- For the same reasons the authors should also avoid to embark on therapeutic speculations.
- The authors state that they analyzed 475 serum samples at different time points (0, 6, 12 and 24 months), however they do not indicate later on how they used the time point a 6 months. Please clarify.
- It is not clear why the confirmative analysis of free serum hemoglobin using ELISA has only been performed in 20 SPMS patients. It would have been advantageous and the authors a strongly encouraged to determine free serum hemoglobin in all 475 serum to see if increased free serum hemoglobin as determined by ELISA was also correlated with increased brain atrophy.
- The Discussion should also reflect still existing uncertainties in a more comprehensive manner.

 

**Minor comments**

- In Figure 3, p-values (corrected for multiple comparisons) of group differences should be included.
- In Figure legend 3 it is mentioned that “...Log [haemoglobin] by ELISA was significantly correlated with log(peak intensity): r = 0.52; p = 0.02; linear regression...”. For better understanding and reading this information should be presented by a separate scatter plot.

We have read this submission. We believe that we have an appropriate level of expertise to confirm that it is of an acceptable scientific standard, however we have significant reservations, as outlined above.

## References

[1] AltinozMAOzcanEMInceBGuloksuzS: Hemoglobins as new players in multiple sclerosis: metabolic and immune aspects. *Metab Brain Dis*.2016;31(5) : 10.1007/s11011-016-9845-7 983-92 10.1007/s11011-016-9845-7 27234993

[2] KhalilMLangkammerCPichlerAPinterDGattringerTBachmaierGRopeleSFuchsSEnzingerCFazekasF: Dynamics of brain iron levels in multiple sclerosis: A longitudinal 3T MRI study. *Neurology*.2015;84(24) : 10.1212/WNL.0000000000001679 2396-402 10.1212/WNL.0000000000001679 25979698

[3] KhalilMRiedlbauerBLangkammerCEnzingerCRopeleSStojakovicTScharnaglHCuleaVPetzoldATeunissenCArchelosJJFuchsSFazekasF: Cerebrospinal fluid transferrin levels are reduced in patients with early multiple sclerosis. *Mult Scler*.2014;20(12) : 10.1177/1352458514530020 1569-77 10.1177/1352458514530020 24777275

[4] KhalilMRennerALangkammerCEnzingerCRopeleSStojakovicTScharnaglHBachmaierGPichlerAArchelosJJFuchsSSeifert-HeldTFazekasF: Cerebrospinal fluid lipocalin 2 in patients with clinically isolated syndromes and early multiple sclerosis. *Mult Scler*.2016;22(12) : 10.1177/1352458515624560 1560-1568 10.1177/1352458515624560 26762671

[5] RopeleSde GraafWKhalilMWattjesMPLangkammerCRoccaMARoviraAPalaceJBarkhofFFilippiMFazekasF: MRI assessment of iron deposition in multiple sclerosis. *J Magn Reson Imaging*.2011;34(1) : 10.1002/jmri.22590 13-21 10.1002/jmri.22590 21698703

# Peer review: Referee response for version 1

**DOI:** 10.21956/wellcomeopenres.10742.r17825

**Published:** 2016-11-21

**Authors:** George Harauz, Vladimir V. Bamm

**Affiliations:** 1 Department of Molecular and Cellular Biology, University of Guelph, Guelph, ON, Canada

**Summary** – This article describes a proteomics analysis of serum proteins derived from 140 patients with secondary progressive multiple sclerosis (MS), 20 healthy adult volunteers, 20 patients with human T‐lymphotropic virus (HTLV‐1) causing symptoms resembling spinal MS, and 20  asymptomatic HTLV‐1 carriers. Half of the MS patients were undergoing treatment with simvastatin,  a drug used to lower blood cholesterol and shown to have immunomodulatory and anti-inflammatory properties. Protein profiling of sera was achieved by mass spectrometry of 475 serum samples collected at 0, 12, and 24 months. (A 6‐month time point is mentioned in some places and is queried below). The MS patients had concurrent MRI scans at 0, 12, and 25 months to measure whole brain volume (BBSI – brain boundary shift integral), presumably amongst other measures. Serum samples were “enriched” and analysed by 1D SDS‐PAGE followed by LC‐MS/MS of in gel digested protein. Free haemoglobin (Hb) levels were assessed by ELISA, and activity of lactate dehydrogenase (LDH), an indicator of general tissue damage, and particularly of haemolysis was measured. The proteomics analysis suggested that a 15.1‐kDa protein peak correlated with the rate of brain atrophy in seemingly all MS patients, regardless of treatment regime. Following protein enrichment, 15.1‐kDa and 15.9‐kDa peaks were observed and confirmed to represent the α‐ and β-chains of haemoglobin, respectively. In all MS patients, levels of both free Hb chains and of LDH activity were elevated compared to all controls. The results are consistent with the idea that Hb is released into serum by chronic and low‐grade intravascular haemolysis, with subsequent  translocation into the CNS where it has great potential to cause oxidative damage.    

**Comments on title and abstract** –

1. We suggest that the word “associated” needs to be substituted by “correlated”
2. Conclusions in the abstract must be linked to the objectives of the study rather than be a  speculative claim.    

**Comments on study design and data interpretation** – Several points require clarification, in our view.    

1. There were 140 patents, and 60 controls (3 groups of 20). So the total number is supposed to be  200 serum samples per time point. What are the other 275 samples? The question of sample numbers, both of patients and controls, arises again later when 138 patients are mentioned. Additionally, a valuable control could be a group of patients with another neurodegenerative disease characterised by brain atrophy.    
2. The 6‐month time point was not mentioned in the paragraph describing the study design, and there were no results reported for it.
3. For protein profiling by SELDI‐TOF mass spectrometry, after the 1:10 serum dilution, one would  expect signal suppression effect on the lower abundance proteins. To remove this effect it would have been advised to fractionate the serum first, and then use protein chip arrays. Since the authors did not fractionate the sera, it is not possible to rule out other lower‐abundance markers, such as hemopexin, as we indicate next.
4. The Top 12 Protein Depletion Spin Columns are a good way to partially fractionate the serum or to enrich the protein of interest. However, several very important proteins (haptoglobin, transferrin, and Apo AI) related to iron homeostasis will be removed by this procedure. In the context of this study, it is important to see the specific expression patterns of haptoglobin, hemopexin, and HO‐1 since they represent different levels of defence mechanisms against extracellular Hb. Also, it could be beneficial to try and correlate different haptoglobin phenotypes with BBSI.
5. In the same vein, the ELISA kit will detect extracellular Hb from two sources: free Hb and haptoglobin‐bound Hb. The latter form could have been removed by the spin column that was used for protein enrichment.
6. Why and how were only 20 patients selected for ELISA?    

**Comments on discussion** – We believe that the Discussion can be augmented to give a broader picture as follows.
** **

1. Association versus correlation of brain volume and Hb levels. In our opinion the results show correlation between brain volume and free Hb levels and are insufficient to claim that these two factors are “associated” per se.
2. In reference to [(Bamm and Harauz 2014)], the authors state that “neuropathological evidence does not show frequent or widespread extravascular erythrocytes in the MS brain”. This statement is not strictly correct. Firstly, in [(Bamm and Harauz 2014)] we did not suggest that chronic extravasation of red blood cells is an exclusive source of the abnormal iron deposits. In fact, we said:  “Any type of seemingly minor yet chronic cerebrovascular abnormality and/or damage to the blood– brain barrier, … , can potentially lead to **intravascular hemolysis**, or to extravasation of erythrocytes and extravascular hemolysis”. Secondly, as we reviewed recently [(Bamm et al. 2016)], extracellular Hb could arise from blood extravasation due to capillary and venous micro‐hemorrhages, which ARE being documented in MS lesions, as possibly are cerebral microbleeds (CBMs) [(Zivadinov *et al.* 2016)]. Such events can be difficult to detect at the histological level, especially in the early stages of lesion formation that can arise from molecular dysfunction rather than gross structural damage. The  potential molecular mechanisms of Hb toxicity in myelin are described in a subsequent experimental  paper [(Bamm *et al.* 2015)] and do not rely on large‐scale iron deposition. The damage inflicted by free Hb can arise at many different levels and per se will not necessarily be associated with the number of extravasated erythrocytes in MS tissue.
3. In reference to erythrocyte fragility in MS, we suggest also citing one of the earlier papers to indicate that this is an old idea that has been insufficiently explored [(Caspary *et al.*1967)], and two newer papers that describe the association of haemoglobin variants with MS severity [(Altinoz *et al.* 2016; Ozcan *et al.* 2016)].
4. It is not clear how the finding of higher free Hb in the sera of MS patients can be explained. The  authors reported that the mean erythrocytes count and total blood Hb did not differ between MS patients and control groups. However, the MS patients were characterized by the increased presence of free Hb. If a simple formula for total Hb is: Hb within the erythrocytes + cell‐free Hb, and the erythrocyte count was similar, then the amounts of cell‐free Hb should be similar. Is the morphology of erythrocytes from MS patients and controls different? Perhaps the erythrocytes from MS patients were hypochromic (less intracellular Hb)? We believe that the authors should clarify this  point.  
5. The first line of defense against extracellular haemoglobin is haptoglobin (Hpt). We have determined recently that the frequency of the hpt1 allele is lower in Australia, and in European and North American countries with a high reported prevalence of MS [(Bamm *et al.* 2016)]. We also recommended an epidemiological study to evaluate the potential association of Hpt phenotype with disease severity and/or comorbidity with cardiovascular disorders, as is being done for Parkinsonism  [(Costa‐Mallen *et al.* 2015; Delanghe *et al.* 2016; Costa‐Mallen *et al.* 2016)]. It should also be pointed out that cardiovascular disorders have been found to be associated with disease severity and brain  atrophy in MS [(Kappus *et al.* 2016)]. This latter reference is certainly relevant to the Discussion of this current study.

We have read this submission. We believe that we have an appropriate level of expertise to confirm that it is of an acceptable scientific standard, however we have significant reservations, as outlined above.

## References

[1] AltinozMAOzcanEMInceBGuloksuzS: Hemoglobins as new players in multiple sclerosis: metabolic and immune aspects. *Metab Brain Dis*.2016;31(5) : 10.1007/s11011-016-9845-7 983-92 10.1007/s11011-016-9845-7 27234993

[2] BammVVGeistAMHarauzG: Correlation of geographic distributions of haptoglobin alleles with prevalence of multiple sclerosis (MS) - a narrative literature review. *Metab Brain Dis*.2016; 10.1007/s11011-016-9923-x 10.1007/s11011-016-9923-x 27807673

[3] BammVVHarauzG: Hemoglobin as a source of iron overload in multiple sclerosis: does multiple sclerosis share risk factors with vascular disorders?. *Cell Mol Life Sci*.2014;71(10) : 10.1007/s00018-014-1570-y 1789-98 10.1007/s00018-014-1570-y 24504127PMC11113400

[4] BammVVLanthierDKStephensonELSmithGSHarauzG: In vitro study of the direct effect of extracellular hemoglobin on myelin components. *Biochim Biophys Acta*.2015;1852(1) : 10.1016/j.bbadis.2014.10.009 92-103 10.1016/j.bbadis.2014.10.009 25463632

[5] CasparyEASewellFFieldEJ: Red blood cell fragility in multiple sclerosis. *Br Med J*.1967;2(5552) :610-1 602559910.1136/bmj.2.5552.610PMC1842120

[6] Costa-MallenPZabetianCPAgarwalPHuSCYearoutDSamiiALeverenzJBRobertsJWCheckowayH: Haptoglobin phenotype modifies serum iron levels and the effect of smoking on Parkinson disease risk. *Parkinsonism Relat Disord*.2015;21(9) : 10.1016/j.parkreldis.2015.07.006 1087-92 10.1016/j.parkreldis.2015.07.006 26228081PMC4554997

[7] Costa-MallenPZabetianCPAgarwalPHuSCYearoutDSamiiALeverenzJBRobertsJWCheckowayH: Response to the letter. *Parkinsonism Relat Disord*.2016;22: 10.1016/j.parkreldis.2015.10.021 110-1 10.1016/j.parkreldis.2015.10.021 26708126PMC5096650

[8] DelangheJRDe BuyzereMLSpeeckaertMMLangloisMR: Haptoglobin phenotype and Parkinson disease risk. *Parkinsonism Relat Disord*.2016;22(1) : http://dx.doi.org/10.1016/j.parkreldis.2015.10.020 108-109 10.1016/j.parkreldis.2015.10.020 26708125

[9] KappusNWeinstock-GuttmanBHagemeierJKennedyCMeliaRCarlERamasamyDPChernevaMDurfeeJBergslandNDwyerMGKolbCHojnackiDRamanathanMZivadinovR: Cardiovascular risk factors are associated with increased lesion burden and brain atrophy in multiple sclerosis. *J Neurol Neurosurg Psychiatry*.2016;87(2) : 10.1136/jnnp-2014-310051 181-7 10.1136/jnnp-2014-310051 25722366

[10] OzcanMEInceBKaradeliHHGedikbasiAAsilTAltinozMA: Higher minor hemoglobin A2 levels in multiple sclerosis patients correlate with lesser disease severity. *Neuropsychiatr Dis Treat*.2016;12: 10.2147/NDT.S109954 2033-8 10.2147/NDT.S109954 27578976PMC4998019

[11] ZivadinovRRamasamyDPBenedictRRPolakPHagemeierJMagnanoCDwyerMGBergslandNBertolinoNWeinstock-GuttmanBKolbCHojnackiDUtriainenDHaackeEMSchweserF: Cerebral Microbleeds in Multiple Sclerosis Evaluated on Susceptibility-weighted Images and Quantitative Susceptibility Maps: A Case-Control Study. *Radiology*.2016; 10.1148/radiol.2016160060 160060 10.1148/radiol.2016160060 27308776

# Peer review: Referee response for version 1

**DOI:** 10.21956/wellcomeopenres.10742.r17609

**Published:** 2016-11-18

**Authors:** Hans Lassmann, Simon Hametner

**Affiliations:** 1 Center for Brain Research, Medical University of Vienna, Vienna, Austria

This is a very interesting study providing convincing evidence for an association between the serum level increases of free haemoglobin with the extent of brain atrophy progression, determined by MRI, in secondary progressive multiple sclerosis. This was originally observed by the authors using an unbiased proteomics approach, aimed at determining potential serum biomarkers for disease progression. The patient collective derives from a well-controlled clinical trial investigating the effect of simvastatin on the rate of brain atrophy progression in secondary progressive MS. Having found this association, the authors then confirmed free hemoglobin increase in SPMS patients by an independent approach using ELISA.

 The study is very well performed, based on a sound and innovative technology and the results have major implications for the understanding of the neurodegenerative process in MS. Interestingly, the association between haemoglobin serum level increase and atrophy rate occurred independently from the effect of simvastatin treatment. Thus increased haemoglobin in the serum may contribute to the neurodegenerative process, but there are other mechanisms additionally involved. By showing also increased levels of serum LDH the authors further support the concept that there is a low degree of hemolysis in the peripheral blood of MS patients, which however is not reflected in reduction of erythrocyte counts, haematocrit or total blood haemoglobin. However, it would also be interesting to address whether there is an association between actual free hemoglobin levels at any of the single time points (not their change over time), as determined by mass spectrometry, and the observed brain atrophy rates. Authors only show a positive association of free hemoglobin increase with brain atrophy levels, which might have a different interpretation than what the authors conclude. Furthermore, the quantitative ELISA data are not linked with MRI brain atrophy rates. Were the 20 MS patients included in the ELISA quantification taken from the big cohort of the proteomics part of the study? Then MRI data would be available, and the analysis could be easily performed.

 There is good indirect evidence that iron loading of the brain may be involved in neurodegeneration in MS through the amplification of oxidative injury. So far, the mechanisms how the human brain is loaded with iron during aging and in the disease process of MS is not fully understood. This study indicates that heme iron, derived from free circulating haemoglobin, may be of major importance in brain iron accumulation in the course of multiple sclerosis. Free hemoglobin, presumably bound to the haemoglobin scavenger haptoglobin, might diffuse into the brain via a partially compromised blood-brain barrier and might lead to iron accumulation within MS basal ganglia and microglia at edges of chronic lesions. The key question, which however remains unresolved here, concerns the cause of increased free haemoglobin in the blood of MS patients. Interestingly, it has already been shown in the late 1960ths that erythrocyte diameters are larger in MS patients (J. Prineas,1968) and that there is a higher fragility of erythrocytes in comparison to controls, which may result in liberation of haemoglobin (E.A. Caspary *et al.*,1967). These observations together with more recent findings, which relate them to other disturbances of iron homeostasis in MS, have been recently discussed in a comprehensive review article (Altinoz *et al.,* 2016). Whether the increased erythrocyte fragility is a genuine metabolic problem or a consequence of a general systemic chronic pro-inflammatory environment in the MS patients is currently unresolved.

We have read this submission. We believe that we have an appropriate level of expertise to confirm that it is of an acceptable scientific standard.

## References

[1] AltinozMAOzcanEMInceBGuloksuzS: Hemoglobins as new players in multiple sclerosis: metabolic and immune aspects. *Metab Brain Dis*.2016;31(5) : 10.1007/s11011-016-9845-7 983-92 10.1007/s11011-016-9845-7 27234993

[2] CasparyEASewellFFieldEJ: Red blood cell fragility in multiple sclerosis. *Br Med J*.1967;2(5552) :610-1 602559910.1136/bmj.2.5552.610PMC1842120

[3] PrineasJ: Red blood cell size in multiple sclerosis. *Acta Neurol Scand*.1968;44(1) :81-90 570698610.1111/j.1600-0404.1968.tb07445.x
